# Analysis of miR-9-5p, miR-124-3p, miR-21-5p, miR-138-5p, and miR-1-3p in Glioblastoma Cell Lines and Extracellular Vesicles

**DOI:** 10.3390/ijms21228491

**Published:** 2020-11-11

**Authors:** Alja Zottel, Neja Šamec, Ana Kump, Lucija Raspor Dall’Olio, Pia Pužar Dominkuš, Rok Romih, Samo Hudoklin, Jernej Mlakar, Daniil Nikitin, Maxim Sorokin, Anton Buzdin, Ivana Jovčevska, Radovan Komel

**Affiliations:** 1 Medical Centre for Molecular Biology, Institute of Biochemistry, Faculty of Medicine, University of Ljubljana, 1000 Ljubljana, Slovenia; neja.samec@mf.uni-lj.si (N.Š.); ana.kump16@gmail.com (A.K.); lucijaraspor@gmail.com (L.R.D.); pia.puzar-dominkus@mf.uni-lj.si (P.P.D.); ivana.jovcevska@mf.uni-lj.si (I.J.); 2 Jožef Stefan International Postgraduate School, 1000 Ljubljana, Slovenia; 3 Institute of Cell Biology, Faculty of Medicine, University of Ljubljana, 1000 Ljubljana, Slovenia; rok.romih@mf.uni-lj.si (R.R.); samo.hudoklin@mf.uni-lj.si (S.H.); 4 Institute of Pathology, Faculty of Medicine, University of Ljubljana, 1000 Ljubljana, Slovenia; jernej.mlakar@mf.uni-lj.si; 5 Shemyakin-Ovchinnikov Institute of Bioorganic Chemistry, Russian Academy of Sciences, Moscow 117997, Russia; nikitin@oncobox.com (D.N.); buzdin@oncobox.com (A.B.); 6 Oncobox ltd., Moscow 121205, Russia; sorokin@oncobox.com; 7 Laboratory of Clinical and Genomic Bioinformatics, I. M. Sechenov First Moscow State Medical University, Moscow 119146, Russia; 8 Moscow Institute of Physics and Technology (National Research University), Moscow region 141700, Russia; 9 OmicsWay Corp., Walnut, CA 91789, USA

**Keywords:** glioblastoma, biomarkers, exosomes, small extracellular vesicles, miRNA, miR-9-5p, miR-21-5p, miR-124-3p, miR-138-5p, vimentin

## Abstract

Glioblastoma (GBM), the most common primary brain tumor, is a complex and extremely aggressive disease. Despite recent advances in molecular biology, there is a lack of biomarkers, which would improve GBM’s diagnosis, prognosis, and therapy. Here, we analyzed by qPCR the expression levels of a set of miRNAs in GBM and lower-grade glioma human tissue samples and performed a survival analysis *in silico*. We then determined the expression of same miRNAs and their selected target mRNAs in small extracellular vesicles (sEVs) of GBM cell lines. We showed that the expression of miR-21-5p was significantly increased in GBM tissue compared to lower-grade glioma and reference brain tissue, while miR-124-3p and miR-138-5p were overexpressed in reference brain tissue compared to GBM. We also demonstrated that miR-9-5p and miR-124-3p were overexpressed in the sEVs of GBM stem cell lines (NCH421k or NCH644, respectively) compared to the sEVs of all other GBM cell lines and astrocytes. *VIM* mRNA, a target of miR-124-3p and miR-138-5p, was overexpressed in the sEVs of U251 and U87 GBM cell lines compared to the sEVs of GBM stem cell line and also astrocytes. Our results suggest *VIM* mRNA, miR-9-5p miRNA, and miR-124-3p miRNA could serve as biomarkers of the sEVs of GBM cells.

## 1. Introduction

Glioblastoma (GBM) is the most common and highly aggressive primary brain tumor, with an annual incidence below 10 (e.g., 3.2 in the USA) per 100,000 people [1,2]. In Europe, the incidence varies from 3 per 100,000 people in Eastern Europe to 5 per 100,000 people in the United Kingdom and Ireland [3]. This is a disease that mainly affects the elderly population, with an average age of patients diagnosed at 64 years [4]. According to the World Health Organization classification, it belongs to grade IV, the most malignant type of glioma [5]. Despite extensive research and attempts to develop appropriate and effective therapies, GBM is still characterized by a poor prognosis and a high mortality rate [6]. In general, 90% of GBM arise *de novo*, while the rest develop from lower-grade glioma [7]. The exact causes of the disease are largely unknown, but high-dose ionizing radiation and rare genetic disorders have been suggested to increase incidence [4]. Diagnosis is usually made with magnetic resonance imaging and confirmed with histology. In most cases, patients are treated surgically and with radiotherapy and chemotherapy with temozolomide [4]. Temozolomide is an alkylating agent that crosses the blood–brain barrier and methylates DNA, leading to cell death [7]. With this aggressive treatment, the survival of GBM patients ranges from 12 to 15 months, while only 9.8% of patients survive more than 5 years after diagnosis [8,9]. There are quite a few reasons why established treatments have no long-term success. One of the main causes is the presence of GBM stem cells, which share characteristics with normal stem cells, such as the expression of common markers, quiescence, and a high ability of self-renewal [7]. In addition, GBM stem cells are highly resistant to drugs, are migratory, and can form a new tumor [7].

To date, only a few glioblastoma biomarkers have entered clinical use. The two major are the methylation status of O-6-methylguanine-DNA methyltransferase (*MGMT*) promotor, and the mutation status of isocitrate dehydrogenase 1 and 2 (*IDH1*, *IDH2*). The methylation of *MGMT* promotor is correlated with a better disease outcome [4], while the status of *IDH* is the basis for GBM’s classification into three subgroups: *IDH*-mutant, wild-type *IDH*, and “not otherwise specified” (NOS) [10,11]. In general, *IDH* mutation is an indicator of a less aggressive form of the disease [1]. Despite recent progress in biomarker discovery, there is still a great shortage of biomarkers that would provide a more accurate insight into the nature or better characterization of the tumor and its prognosis, and on this basis could finally adjust appropriate therapy. Additionally, the disadvantages of current diagnosis are that MRI sometimes cannot distinguish well enough between GBM and other brain tumors, and single biopsies may not represent the overall genetic diversity of a given sample [12].

Newly explored potential biomarkers are various RNA molecules, including miRNAs, which are small (18–25 nucleotide long) non-coding RNAs that bind to the 3′UTR region of the target mRNA [13]. miRNAs are especially attractive because they can regulate the expression of target genes and affect the expression levels of the corresponding proteins [14]. The emerging and promising source of RNA biomarkers are extracellular vesicles (EVs) [12]. EVs were first described as entities for disposing cellular waste, yet are today known as important means of intercellular communication [15]. They are classified as exosomes, microvesicles, apoptotic bodies, and oncosomes [16]. Exosomes are the smallest among EVs (diameter 50–150 nm) and are frequently studied due to the biologically active molecules they contain. They are formed in multivesicular endosomes (MVE), which release its intraluminal vesicles as exosomes when MVE fuse with the cell’s plasma membrane [15]. When exosomes encounter a target cell, they can trigger signaling by binding to specific receptors, fusing with the membrane, or undergoing endocytosis [15].

Exosomes also carry a variety of macromolecules such as specific proteins and RNAs, making them a suitable source of biomarkers in body fluids. Their internal cargo may contribute to various characteristic processes of malignancy, such as increased cell viability, chemoresistance, angiogenesis, and the activation of malignant signaling pathways such as the signal transducer and activator of transcription (STAT) and sex-determining region Y (SRY)-box 2 (SOX2) pathways [16,17]. The analysis of RNA molecules present in the exosomes has various advantages compared to free RNA, such as protection from RNAse degradation resulting in longer stability and the detection of small amounts of RNAs that might otherwise go unnoticed [12]. On the other hand, the main disadvantages of this analysis are the small number of copies of specific gene transcripts and the difficulty in choosing the right reference for normalization [12].

As the field of research on extracellular vesicles is relatively new, no agreement has been reached on biomarkers specific to different subtypes of extracellular vesicle [18]. Therefore, as suggested by the Minimal Information of Extracellular Vesicle Studies 2018 [18], vesicles in our study are referred to as small extracellular vesicles (sEVs) (<200 nm) instead of exosomes.

In this study, we elucidated the differences in the expression levels of selected miRNAs and their target genes in different human-derived GBM samples. We examined the expression levels of miRNAs between GBM, lower-grade glioma, and reference brain tissue samples. Next, we examined the expression level of miRNAs and mRNAs in sEVs derived from GBM cell lines and astrocytes. Finally, we also performed a pathway analysis to investigate in which signaling pathway a particular candidate miRNA is involved.

## 2. Results

### 2.1. Selection of a Set of miRNAs (miR-9-5p, miR-21-5p, miR-124-3p, miR-138-5p, and miR-1-3p) That Target VIM, NCL, NAP1L1, FREM2, and SPRY1 Genes

Candidate miRNAs were selected based on their target genes, *vimentin* (*VIM*), *nucleolin* (*NCL*), *nucleosome assembly protein-1 like-1* (*NAP1L1*), *FRAS1-related extracellular matrix protein 2* (*FREM2*), and *sprouty RTK signaling antagonist 1* (S*PRY1*) [6,19,20,21], which were previously associated with GBM in our studies. The selection was based on the prediction of the high probability and consistency of miRNA candidates among TargetScan [22], miRDB [23,24], and microT-CDS datasets [25,26]. The selection of miRNAs is shown in Table 1.

miR-9-5p targets *NAP1L1* and *FREM2* genes, which matched all tested datasets (TargetScan, miRDB, and microT-CDS). miR-21-5p targets *SPRY1* gene and miR-124-3p targets *SPRY1*, *NAP1L1*, and *VIM* genes, with only *NAP1L1* and *VIM* genes being consistent among all three datasets. Furthermore, miR-138-5p targets *VIM* and *NCL* genes, with *VIM* gene as a target consistent only between TargetScan and miRDB, while *NCL* gene as a target was detected only by microT-CDS. On the other hand, miR-1-3p was described to target *NCL* gene according to all three databases.

According to TargetScan, miR-9-5p binds the 672–679 position of 3′UTR *FREM2* and the 347–353 position of 3′UTR *NAP1L1*. miR-21-5p binds the 415–422 position 3′UTR of *SPRY1*. miR-124-3p binds the 928–935 position of 3′UTR of *SPRY1*, the 403–409 position of 3′UTR *NAP1L1*, and the 81–87 position of 3′UTR of *VIM*. miR-138-5p binds the 32–38 position of 3′ UTR *VIM* and miR-1-3p binds the 90–97 position of 3′UTR of *NCL*.

### 2.2. miR-9-5p, miR-21-5p, miR-124-3p, and miR-1-3p Are Involved in Cancer and Brain-Specific Processes

**Figure 1:**
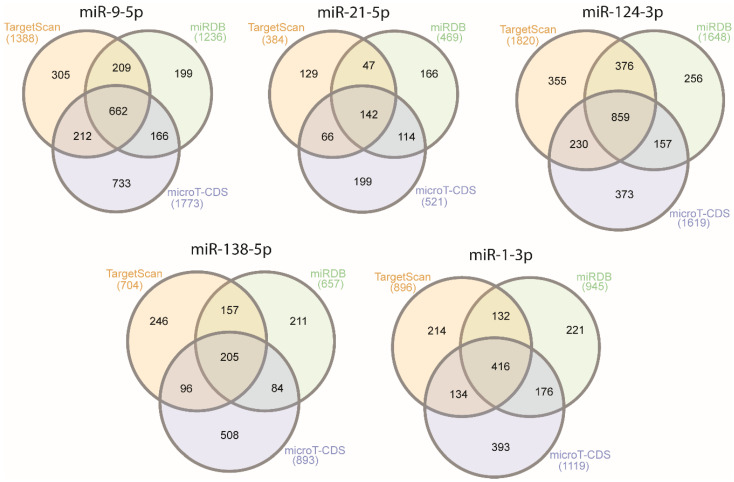
Venn diagrams of number of candidate miRNA target genes obtained from the TargetScan, miRDB, and microT-CDS datasets.

For further insight into the function of candidate miRNAs, we identified pathways associated with candidate miRNAs. We first determined the target genes using the TargetScan [22], miRDB [23,24], and microT-CDS [25,26] datasets. Venn diagrams [28] of common target genes from all three datasets are presented in Figure 1. The list of genes is available in Supplementary Table S1.

The roles of miRNAs were analyzed by an Over-Representation Analysis of common target genes obtained from TargetScan, miRDB, and microT-CDS, using WebGestalt [29,30,31,32] and the Kyoto Encyclopedia of Genes and Genomes (KEGG) [33], Panther [34], and Reactome [35] databases. The pathways for each miRNA are presented in Table 2, along with their false-discovery rate (FDR). Only pathways with a false-discovery rate <0.05 are presented. In Supplementary Figures S1–S3, the protein connections for two pathways for miR-9-5p and miR-21-5p are presented. The protein connections were analyzed by STRING [36]. Only experimentally validated interconnections are presented. Supplementary Figure S1 shows the interconnections of proteins involved in the RAS pathway. The figure shows that miR-9-5p inhibits genes including *MAP2K7*, *MAP3K1*, *SHC1*, and *PIK3CP*, and in the ErbB pathway (Supplementary Figure S2) it inhibits genes including *PAK4, MAP2K7, GSK3B*, and *SOS1*. Supplementary Figure S3 shows the involvement of miR-21-5p in the RAS pathway, inhibiting genes including *GAB1*, *FASLG*, *TIAM1*, and its involvement in the tyrosine kinase pathway, inhibiting genes including *SPRY2*, *FGF18*, *PAG1*, and *STAT3*.

Most of the miRNAs analyzed are potentially involved in cancer, either specific types such as hepatocellular carcinoma (miR-9-5p), renal cell carcinoma (miR-1-3p), and thyroid cancer (miR-1-3p), or specific processes associated with cancer (miR-9-5p, miR-21-5p, miR-124-3p, and miR-1-3p). Moreover, miR-124-3p and miR-1-3p were related to the brain-specific processes of axon guidance, and miR-9-5p was strongly involved in the Ras, neurotrophin, and ErbB pathways. Likewise, miR-21-5p was involved in the Ras signaling pathway, as well as in the NTRK, BMP, receptor tyrosine-kinase, and PI3K: FGF pathways. For miR-138-5p, no pathway with an FDR lower than 0.05 was detected by Reactome, Panther, or KEGG.

### 2.3. miR-124-3p and miR-138-5p Are Overexpressed in Reference Brain Tissue Compared to GBM

**Figure 2:**
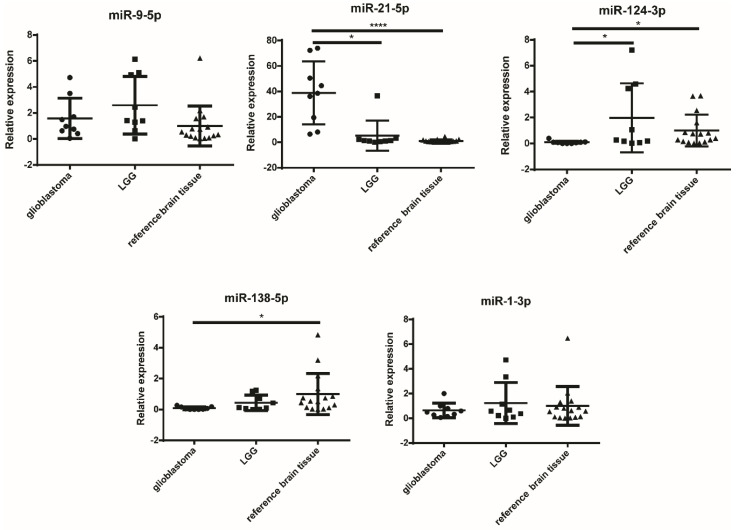
Relative expression levels of miRNA in glioblastoma, LGG, and reference brain tissues. * *p* < 0.05, and **** *p* < 0.0001. Results are presented as mean, and standard deviation as error bars. LGG—lower-grade glioma.

Next, we were interested whether the candidate miRNAs are expressed differently in different grades of glioma (GBM and lower-grade gliomas (LGG)) and reference brain tissues. The results of the experiment are graphically presented in Figure 2, and the results of the statistical analysis are given in Table 3.

miR-21-5p was significantly overexpressed in GBM compared to both LGG (*p* = 0.0146) and reference brain tissue (*p* < 0.0001). On the other hand, miR-124-3p was significantly overexpressed in reference brain tissue compared to GBM (*p* = 0.0178) and also in LGG compared to GBM (*p* = 0.0278). Likewise, miR-138-5p was also overexpressed in reference brain tissue compared to GBM (*p* = 0.0143). In contrast, for miR-9-5p and miR-1-3p no differences in tissue expression levels were detected.

### 2.4. Higher Expression Levels of miR-9-5p and miR-138-5p Are Correlated to the Shorter Survival of GBM Patients Carrying IDH Mutation

**Figure 3:**
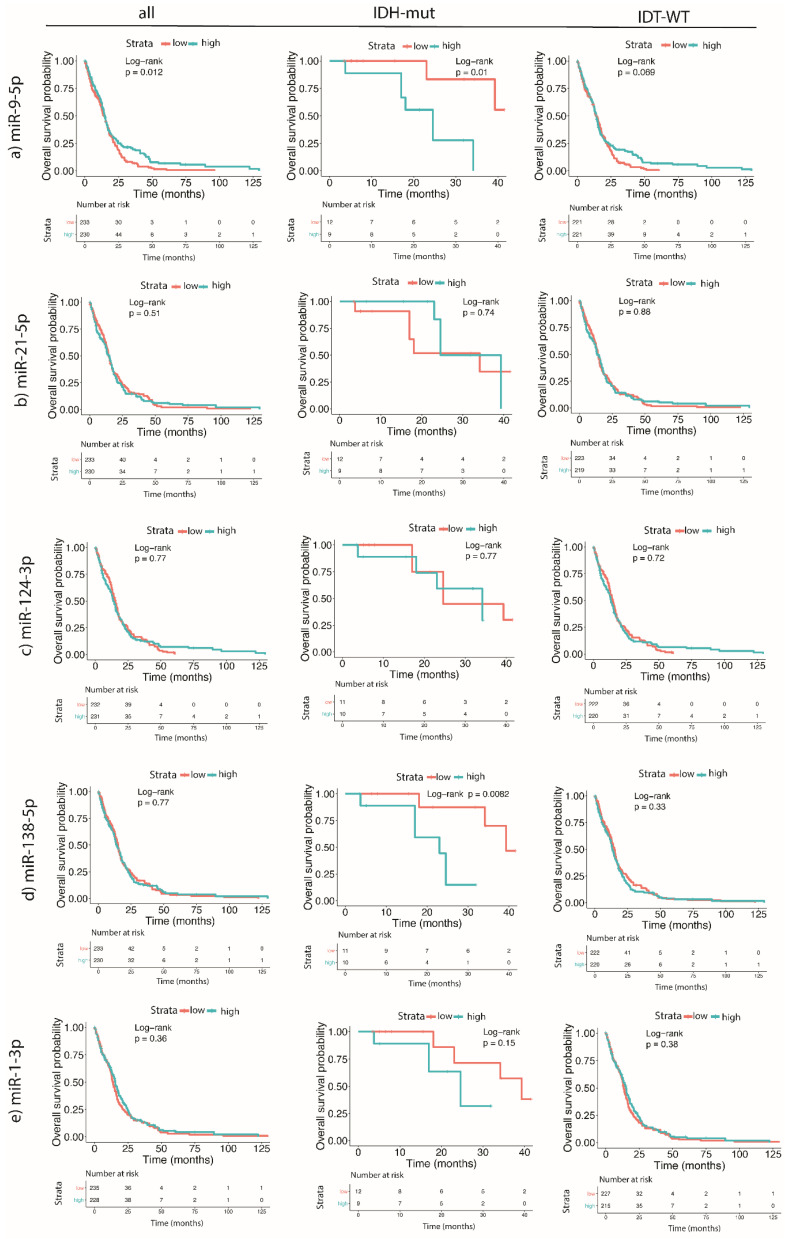
Survival analysis in GBM patients based on the expression levels of miRNAs (miR-9-5p, miR-21-5p, miR-124-3p, miR-138-5p, and miR-1-3p) and *IDH* mutation status in GBM tumor tissues. (**a**) miR-9-5p, (**b**) miR-21-5p, (**c**) miR-124-3p, (**d**) miR-138-5p, (**e**) miR-1-3p. GBM—glioblastoma.

We wanted to investigate whether the expression of candidate miRNAs was associated with the survival of GBM patients, as this would mean that potential miRNA could be used as a prognostic biomarker. The results are graphically presented in Figure 3. The analysis was performed *in silico* using the TCGA database.

The results of the survival analysis showed that the higher expression of miR-9-5p in GBM tissues correlated with a shorter median survival of *IDH*-mutant GBM patients, whereas in all patients, regardless of the *IDH* mutation status, a higher miR-9-5p was linked to better survival. Similarly, a high expression of miR-138-5p was linked to worse survival in GBM patients carrying an *IDH* mutation. The expression of other tested miRNAs (miR-21-5p, miR-124-3p, or miR-1-3p) did not affect mean differences in survival.

### 2.5. Characterization of sEVs

**Figure 4:**
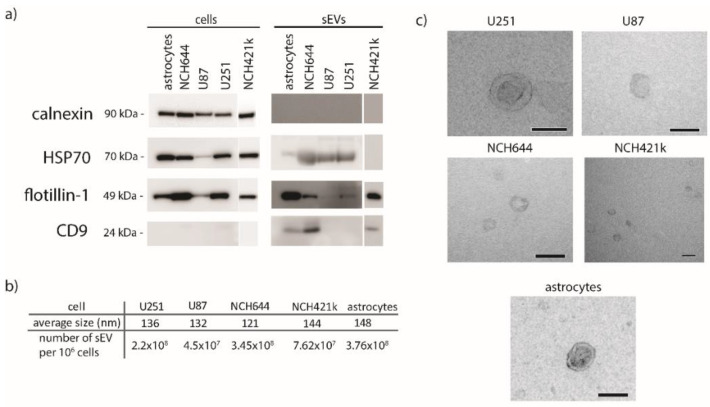
Molecular characterization of sEVs isolated from normal astrocytes and GBM cell lines. (**a**) Western blot of cell and sEV lysates. Antibodies directed against proteins enriched in sEVs (HSP70, flotillin-1, CD9) and against a marker for endoplasmic reticulum (calnexin) were used. (**b**) Mean size of sEVs was determined by nanoparticle-tracking analysis. (**c**) Transmission electron microscopy images of sEVs. Scale bare is 100 nm. sEVs—small extracellular vesicles.

The sEVs were isolated from the U251 and U87 cell lines, from the NCH644 and NCH421k glioblastoma stem cell lines and astrocytes as a reference. NCH644 and NCH421k were characterized by flow cytometry, detecting CD133 on their surface (Supplementary Figure S4). The results show that both NCH644 and NCH421k have a high expression of CD133 (61–66%), while the expression of CD133 on U251, U87, and astrocytes was almost absent. The sEVs isolated from GBM cell lines and astrocytes were characterized by Western blot, nanoparticle tracking analysis (NTA), and transmission electron microscopy to confirm their identity and ensure that there were no cellular contaminants. The results are presented in Figure 4. The isolated sEVs were positive for proteins commonly enriched in sEVs: heat shock protein 70 (HSP70), flotillin-1, and CD9. The purity of sEV isolates was confirmed by the absence of calnexin, a marker for endoplasmic reticulum (Figure 4a). HSP70 and flotillin-1 were detected in lysates of all cells and sEVs, except in the sEVs of NCH421k (HSP70). The signal of flotillin in U87 sEVs was very weak, which may indicate that it is present in a lesser extent in U87 cells. Furthermore, CD9 was detected only in the sEVs of astrocytes and in the sEVs of the GBM stem cell lines NCH644 and NCH421k. The size of sEVs was determined by nanoparticle-tracking analysis (NTA). The mean diameter of all sEVs was between 100 and 150 nm (Figure 4b), which is expected because the cell medium was filtered through 220 nm-pore-size filters. The smallest sEVs were derived from the NCH644 cell line and measured 121 nm in diameter, while the largest were those from astrocytes (148 nm). Additionally, the number of sEVs secreted by a million cells was calculated. Astrocytes and NCH644 secreted the highest number of sEVs (3.76 × 10^8^ per million cells and 3.45 × 10^8^ per million cells), while U87 secreted the least number of sEVs (4.5 × 10^7^ per million cells). sEVs were also observed under the electron microscope, and representative images are presented in Figure 4c. All the observed sEVs had a spherical/doughnut-like shape. We conclude that all five investigated cell types release sEVs with distinct characteristics. 

### 2.6. miR-9-5p and miR-124-3p are Overexpressed in the sEVs of NCH421k and NCH644 GBM Stem Cell Lines, Respectively, in Comparison to the sEVs of GBM Cell Lines and Astrocytes

**Figure 5:**
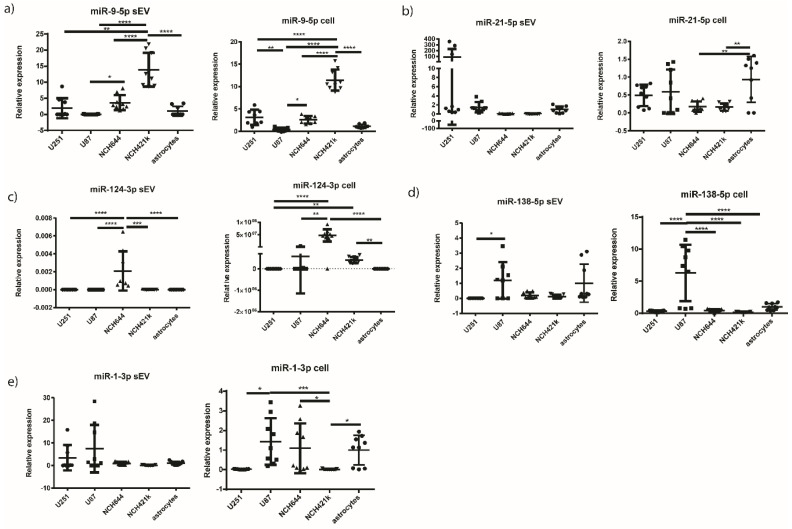
Relative miRNA expression in GBM cell lines and normal astrocytes and in their corresponding sEVs. Results are presented as mean, and standard deviation as error bars. (**a**) miR-9-5p, (**b**) miR-21-5p, (**c**) miR-124-3p, (**d**) miR-138-5p, (**e**) miR-1-3p. * *p* < 0.05, ** *p* < 0.01, *** *p* < 0.001, and **** *p* < 0.0001. For the miR-124-3p in sEVs, data normalized to the reference gene are presented, because the expression of miR-124-3p in the sEVs of astrocytes was below the detection threshold. GBM—glioblastoma. sEVs—small extracellular vesicles.

We then performed qPCR on candidate miRNAs (miR-9-5p, miR-21-5p, miR-124-3p, miR-138-5p, and miR-1-3p) from all five cell types (U251, U87, NCH644, NCH421k GBM cell lines and astrocytes as a reference) and corresponding sEVs. The results are presented in Figure 5 and Table 4. It should be noted, however, that the experiment including the NCH421k cell line was conducted separately from the rest of the cells.

miR-9-5p was upregulated in the NCH421k cell line compared to all other GBM cell lines and astrocytes (*p* < 0.0001 for all cells). Similarly, miR-9-5p was upregulated in the sEVs of the GBM stem cell line NCH421k, compared to the sEVs of NCH644, U87, U251, and astrocytes (*p* = 0.0048 for U251 and *p* < 0.0001 for U87, NCH644, U87, and astrocytes). Furthermore, miR-21-5p was found to be significantly overexpressed in astrocytes cells compared to NCH644 (*p* = 0.0052) and NCH421k (*p* = 0.0042), while there were no statistical differences observed between other cells and also between sEVs. For miR-124-3p, it was found to be significantly overexpressed in the both GBM stem cell lines, NCH644 and NCH421k, compared to astrocytes (*p* < 0.0001 and *p* = 0.0075, respectively). In sEVs, the expression of miR-124-3p was significantly higher only in NCH644 compared to all the other cells (*p* < 0.0001 for U251, U87, and astrocytes, *p* = 0.0002 for NCH421k). However, in astrocyte sEVs the miR-124-3p expression level was below the limit of detection (Figure 5). Furthermore, miR-138-5p was overexpressed in the U87 cell line compared to all other cells tested (*p* < 0.0001 for all cells), but in associated sEVs it was overexpressed in U87 sEVs compared to U251 sEVs only (*p* = 0.0301). On the other hand, miR-1-3p was found to be overexpressed in U87 and astrocytes compared to NCH421k (*p* = 0.0007). The results indicate that miR-124-3p may differentiate between glioblastoma stem cell lines (NCH644 and NCH421k) and astrocytes. In addition, miR-124-3p and miR-9-5p could also be considered for further investigation as potential biomarkers for GBM stem cell sEVs.

### 2.7. Vimentin Gene Is Overexpressed in sEVs Originating from U251 and U87 GBM Cell Lines

**Figure 6:**
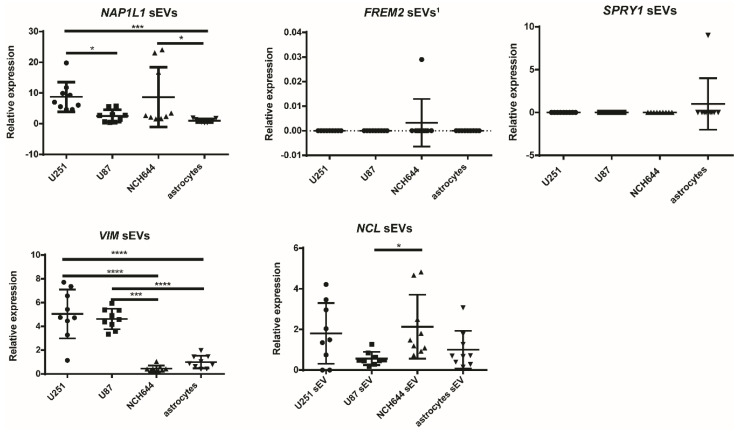
Relative mRNA expression in GBM and normal astrocyte sEVs. Results are presented as mean, and error bars represent standard deviation. * *p* < 0.05, *** *p* < 0.001, and **** *p* < 0.0001. *FREM2* sEVs ^1^—data normalized to reference gene are not presented, as the expression of *FREM2* in sEVs of astrocytes was under the detection threshold. GBM—glioblastoma. sEVs—small extracellular vesicles.

Finally, we investigated whether the target genes of the selected miRNAs—*NAP1L1, FREM2, SPRY1, VIM*, and *NCL*—that have been associated with GBM in our previous studies [6,19,20,21] were also present in the sEVs of different cell types and showed any particular expression pattern in GBM-isolated sEVs compared to normal astrocyte cells. qPCR was used for analysis. The results are presented in Figure 6, and the results of the statistical analysis in Table 5. NCH421k sEVs were not included in this study, as reference genes could not be detected in any of the NCH421k samples.

*VIM* gene had a significantly higher expression in the U251 and U87 cell lines’ sEVs compared to the NCH644 GBM stem cell line sEVs (*p* < 0.0001 and *p* = 0.0003, respectively) and normal astrocyte sEVs (*p* < 0.0001), while the *NAP1L1* gene expression was significantly higher in sEVs from U251 and NCH644 compared to those of astrocytes (*p* = 0.0002 and *p* = 0.0300, respectively). Here, the mRNA expression was also higher in U251 sEVs compared to U87 (*p* = 0.0467). On the other hand, there was no differential expression of *NCL, FREM2*, and *SPRY1* genes in the sEVs of cancer cell lines and astrocytes. Because the *FREM2* expression in astrocytes could not be detected, in this case data are presented without normalization to astrocytes. The results suggest that the *VIM* gene expression in sEVs may differ between the U251 and U87 GBM cell lines and normal astrocytes.

We then compared the gene expression in sEVs with the expression in cells types, as previously published [19,21]. The expression profile of *VIM* gene in sEVs followed the expression in cells, while the expression profile of the *NAP1L1*, *NCL*, *SPRY1*, and *FREM2* genes in sEVs did not follow the expression profile of the same genes in different cell types [19,21].

Finally, we compared the expression of selected miRNAs in sEVs with the expression of their target genes in sEVs. *SPRY1* and *FREM2* genes were both negative in the sEVs of all cell types, while miR-9-5p and miR-21-5p expression was detected in all cell types, indicating inverse expression. The *VIM* gene was significantly overexpressed in the sEVs of the U251 and U87 glioblastoma cell lines, while *VIM*-targeting miR-124-3p showed an inverse expression compared to *VIM* itself, as it was downregulated in the sEVs of U251 and U87 compared to the NCH644 stem cell line. Additionally, *VIM*-targeting miR-138-5p had inverse expression in the U251 sEVs compared to astrocytes and stem cell lines (though not significant). In Table 6, the summary of all the results are presented.

## 3. Discussion

In this study, we selected candidate miRNAs that regulate genes associated with GBM, as confirmed by our previous studies. FREM2 and SPRY1 have been proposed as potential new markers of GBM, and have been demonstrated to be significantly upregulated in GBM at the mRNA and/or protein level in GBM cell lines and GBM tissues compared to references (astrocytes or reference non-malignant brain tissue) [6,19]. Furthermore, *VIM, NCL*, and *NAP1L1* were significantly overexpressed in GBM tissues and/or cells compared to the reference [20,21]. Some of the proposed genes have also been implicated in other cancers, for example gastric cancer (*vimentin*) [37], large B-cell lymphoma (*nucleolin*) [38], and pancreatic cancer (*NAP1L1*) [39]. The information about *FREM2* expression in cancer is rather scarce. However, it has been found to be overexpressed in gliosarcoma [40]. *SPRY1*, on the other hand, has been associated with increased malignancy in triple-negative breast cancer [41] and has been shown to be downregulated in epithelial ovarian cancer [42].

The investigated miRNAs were selected on the basis of the datasets TargetScan [22], miRDB [23,24], and microT-CDS [25,26], with a high probability of miRNA–mRNA pair formation. Most of the miRNA–target gene combinations matched between the datasets—namely, miR-9-5p targeting the *NAP1L1* and *FREM2* genes; miR-21-5p regulating the *SPRY1* gene; miR-124-3p regulating the *SPRY1*, *NAP1L1*, and *VIM* genes; and miR-1-3p regulating the *NCL* gene. Discrepancy was observed in the case of miR-138-5p, which regulates the *VIM* gene, as predicted by TargetScan and miRDB, and the *NCL* gene, as predicted by microT-CDS. Of these two, *VIM* has already been described as a direct target of miR-138 [43,44], while to our knowledge there are no direct experimental reports on *NCL* as a target of miR-138.

The analysis of the miRNA expression in cells showed that miR-9-5p was overexpressed in the GBM NCH421k stem cell line compared to all other GBM cell lines and astrocytes. It was similar to the expression in sEVs, as this miRNA is also overexpressed in the sEVs of the GBM NCH421k stem cell line compared to the sEVs of all other GBM cell lines and astrocytes. Our findings are consistent with the results published by Schraivogel et al., who reported a high expression of miR-9 in CD133+ glioma stem cells [45]. On the other hand, Tezcan et al. [13] found that the miR-9 expression in GBM stem cells was lower compared to the expression in GBM non-stem cells, although not significantly. Our results suggest that miR-9-5p is overexpressed in one type of glioblastoma stem cell line (NCH421k) and their sEVs compared to healthy astrocytes. Our results also show that a higher expression of miR-9-5p in the GBM tissue is associated with a poorer survival (*p* = 0.01) in patients with *IDH*-mutated GBM, whereas in all tissues, regardless of the *IDH* mutation, a higher miR-9-5p expression was associated with better survival (*p* = 0.012). Similar results were obtained by Ben-Hamo et al., who showed with *in silico* analysis that higher miR-9-5p is linked to better survival; however, in this study *IDH* status was not taken into consideration [46]. The analysis of the participation of our selected miRNAs in different cellular pathways (Table 2) showed that miR-9 is strongly involved in the modulation of the Ras, ErbB, neurotrophin signaling, and actin cytoskeleton pathways, which have previously been associated with malignancy [46,47,48,49]. However, to better elucidate the role of miRNAs in a particular pathway, it would be appropriate to establish a knock-down of miRNA and explore the expression of downstream target(s). miR-9 has also been reported as one of the contributors to temozolomide resistance in the GBM stem cells. Specifically, as described by Munoz et al., anti-miR-9 present in exosomes successfully inhibited temozolomide resistance in GBM cells [50]. Therefore, miR-9, selectively packaged in NCH421k sEVs, may mediate and promote temozolomide resistance, as the GBM stem cells are exceptionally resistant to chemotherapy [51]. Nevertheless, the potential role of miR-9 in mediating temozolomide resistance should be further investigated.

Another miRNA that is specific for GBM is miR-21-5p (Figure 2), as the expression of miR-21-5p was significantly overexpressed in GBM compared to LGG (Figure 2) and reference brain tissue. Our results indicate that miR-21-5p, in contrast to miR-9-5p, was significantly downregulated in GBM stem cell lines (both NCH644 and NCH421k) compared to astrocytes. The expression of miR-21 has already been positively associated with a more differentiated phenotype and negatively associated with stemness [52,53]. While miR-21-5p has already been investigated as a marker of GBM present in the sEVs of cerebrospinal fluid [54], its presence in other body fluids remains to be investigated. Our results, however, do not indicate differences in the miRNA expression in sEVs of different cell types. Our pathway analysis (Table 2) shows that miR-21-5p is strongly involved in several pathways such as the Ras, NTRK, bone morphogenic protein, receptor tyrosine kinase, MET/PI3K/AKT, and fibroblast growth factor receptor pathways, which have already been associated with GBM [55,56,57]. The targets of both miRNAs, miR-9-5p and miR-21-5p, include *FREM2* and *SPRY1*, which could not be detected in the sEVs of GBM cell lines, and their expression was inverse to that of miRNA expression, suggesting the inhibition of gene expression.

In contrast to miR-21-5p miRNA, the miR-124-3p and miR-138-5p miRNAs were both underexpressed in GBM compared to reference brain tissue (Figure 2). miR-124-3p was significantly downregulated in GBM compared to reference brain tissue and LGG. However, it was overexpressed in NCH644 and NCH421k cell lines compares to astrocytes, as well as in the sEVs of NCH644 compared to astrocytes, in which the expression was below the detection threshold. The absence of miR-124-3p expression in astrocytes has already been reported [58]. miR-124 has previously been shown to be downregulated in GBM stem cells compared to neural stem cells [59], which may serve as more suitable control cell line. As shown in Table 2, the analysis of signaling pathways demonstrated that miR-124-3p is associated with senescence, a feature attributed to cancer stem cells [60]. However, as described as Jiang et al., miR-124 is linked to the inhibition of self-renewal and mesenchymal transition and a reduction in stemness [61]. Nevertheless, in our study its lower expression in cancer stem cell lines compared to the U251 and U87 GBM cell lines could not be observed. miR-124 has also been described as neuron-specific [62], which is also indicated by the results of our pathway analysis linking miR-124-3p to axon guidance (Table 2). This would also explain the low expression in U251 and U87 GBM cells, as GBM belongs to the group of astrocytomas [5].

The second miRNA, underexpressed in GBM vs. reference brain tissue, was miR-138-5p. *In silico* analysis using data from the TCGA showed that a high expression of miR-138-5p was associated with a worse overall survival in GBM patients carrying an *IDH* mutation. In previous studies, miR-138 has been described in brain tumors as both a tumor suppressor and an oncogene [63,64,65]. WebGestalt by KEGG, Panther, or Reactome with a false detection rate below 0.05 (Table 2) did not detect any signaling pathway associated with miR-138 (Table 5). This is probably due to the fact that miR-138 is a relatively uncharacterized miRNA. Both miRNAs, miR-124-3p and miR-138-5p, target the *VIM* gene, which was found overexpressed in the sEVs of differentiated GBM cell lines compared to the GBM NCH644 stem cell line and astrocytes. The expression of this gene was inversely associated with the expression of miR-124-3p and miR-138-5p, suggesting a malignant role of the *VIM* gene that could be further investigated as a potential glioblastoma biomarker in small extracellular vesicles. *VIM* gene encodes the vimentin protein, which belongs to group III intermediate filaments [66]. In physiological conditions, it is expressed in developing brains and later replaced by other filaments. Brain tumors, on the other hand, express proteins that are characteristic for developing brains, including vimentin [67]. It should be noted that it is one of the markers of epithelial to mesenchymal transition and is highly expressed in mesenchymal cells and strongly associated with GBM malignancy [66,68].

## 4. Materials and Methods

### 4.1. Selection of Candidate miRNAs

Candidate miRNAs were selected based on their interaction with *NCL*, *VIM NAP1L1*, *FREM2*, and *SPRY1* [6,19,20,21] target genes using the TargetScan [22], miRDB [23,24], and microT-CDS datasets [25,26].

### 4.2. Pathway Analysis

First, target genes of each miRNA were chosen based on the TargetScan [22], miRDB [23,24], and microT-CDS datasets [25,26]. Next, using Venn diagram [28], all common target genes of miRNAs were selected. Pathway analysis was performed using WebGestalt 2019 [29,30,31,32], including the Panther, Reactome, and KEGG databases for an Over-Representation Analysis. Moreover, a STRING [36] analysis for a few miRNAs and pathways was performed, showing the experimentally validated interconnections of proteins with a level of medium confidence.

### 4.3. Tissues and Ethical Approval

All tissues were of human origin. Nine glioblastoma and nine lower-grade glioma tissues and 16 reference brain tissues were included in the study. All of the GBM tissues were *IDH* wild type. One of the tissues included in lower-grade glioma group was grade II-III glioma (classification by World Health Organization). The tissues were reviewed by a pathologist. The study was approved by the National Medical Ethics Committee of Republic of Slovenia (document numbers are 92/06/12, 89/04/13, 95/09/15, 0120-196/2017/7, and 0120-190/2018/4). Reference samples were acquired following national legal regulations of the Republic of Slovenia.

### 4.4. miRNA Quantification in Tissues

Total RNA from tissues was extracted using TRIzol reagent following the manufacturer’s recommendations. RNA samples were stored at −80 °C and concentrations were measured with Synergy H4 (Synergy H4 Hybrid Reader, BioTek, Winooski, VT, USA), and the A260/A280 ratio was determined. The miRNA conversion to cDNA and its quantification with qPCR are described in Section 4.9 (Quantification of Cellular and sEV miRNA, and sEV mRNA). The process of miRNA to cDNA synthesis was as follows. First, to remove possible DNA traces RNA was treated with DNase (0.1 μL; Roche, Basel, Switzerland) in 10× DNAse buffer (0.5 μL; Roche) in a total volume of 5 μL. The reaction mixture was incubated for 15 min at 30 °C and 10 min at 75 °C. Then, miRNA was transcribed to cDNA using the TaqMan Advanced miRNA cDNA Synthesis Kit according to the manufacturer’s manual (ThermoFisher Scientific, Waltham, MA, USA). The qPCR mixture consisted of 2.25 μL of cDNA, 0.25 μL of probes, and 2.5 μL of TaqMan Fast Advanced Master Mix (ThermoFisher Scientific) in a total of 5 μL reaction. The settings were as follows: 20 s at 95 °C; 45 cycles of 1 s 95 °C, 20 s 60 °C; hold 4 °C. qPCR was performed using ViiA7 (Applied Biosystems, Life Technologies, Waltham, MA, USA). The experiment was performed with three biological replicates and each biological replicate was tested in triplicate. The reference gene for miRNA was miR-99a-5p, one of the stably expressed miRNAs in glioblastoma [69].

### 4.5. In Silico Survival Analysis

The miRNA expression and survival data were extracted from the OncoLnc portal (http://www.oncolnc.org) [70]. In total, we analyzed data for 484 GBM patients (21 with *IDH* mutation found and 463 wild-type patients). *IDH* mutation status was inferred from annotated vcf files downloaded from the TCGA repository (https://portal.gdc.cancer.gov/repository; National Cancer Institute, Bethesda, MD, USA) [71]. We searched for missense mutations *IDH1* R132H and *IDH2* R172H that passed all quality filters imposed by the TCGA consortium.

A survival analysis of GBM patients was performed using R packages survival (Version 3.1.12) and survminer (Version 0.4.6), Kaplan–Meier curves were drawn using R ggplot2 package (Version 3.3.0).

### 4.6. Cell Lines

U251 and U87 human GBM cell lines (ATCC) were cultured in Eagle’s minimum essential medium (Sigma-Aldrich, St. Louis, MO, USA) supplemented with 5% fetal bovine serum (FBS) (Sigma-Aldrich), 1 mM sodium pyruvate (Sigma-Aldrich), 1% non-essential amino-acids solution (Gibco, Waltham, MA, USA) and 1% antibiotic–antimycotic solution (penicillin/ streptomycin/ amphotericin B; Gibco). The NCH644 and NCH421k human GBM stem cell line (CLS Cell Lines Service GmbH, Eppelheim, Germany) were cultured in Neurobasal medium (Gibco) supplemented with 1% GlutaMax (Gibco), B27 supplement (Gibco), 20 ng/mL EGF (Gibco), 20 ng/mL bFGF (Gibco), 1 U/mL heparin (Sigma-Aldrich), and 1% antibiotic–antimycotic solution (penicillin/streptomycin/amphotericin B; Gibco). Both of the glioblastoma stem cell lines are tumorigenic and highly CD133 positive. U251 and U87 are immortalized cell lines commonly used as a model for research of GBM molecular and cell biology. Human astrocytes (ScienCell) were grown in Astrocyte medium (ScienCell, Carlsbad, CA, USA) supplemented with 2% FBS (ScienCell), 1% astrocyte growth supplement (ScienCell), and 1% penicillin/ streptomycin (ScienCell). They were isolated from cerebral cortex and are highly glial fibrillary acidic protein (GFAP) positive. FBS used in the medium for sEVs isolation was previously centrifuged at 100,000× *g* (MLA-50; Beckman Coulter, Brea, CA, USA) and 4 °C for 24 h and filtered through a 0.22 μm-pore-size filter (cellulose acetate membrane, Corning) to eliminate EVs.

#### CD133 Flow Cytometry

The expression of the surface marker CD133 (Prominin-1) was quantified by flow cytometry. Briefly, 50,000 cells of the cell lines NCH644, NCH421K, U87, U251 and astrocytes were resuspended in 0.02% BSA/PBS, centrifuged at 300× *g* for 10 min and the pellet was resuspended in 0.02% BSA/PBS with 0.25 µg of CD133 antibody (CD133 (Prominin-1) Monoclonal Antibody (TMP4), Alexa Fluor 488, eBioscience™). After 30 min incubation at 4 °C, cells were centrifugated at 300× *g* for 10 min, supernatant was discarded and resuspended in 0.02% BSA/PBS. This step was performed twice. Cells were analyzed by flow cytometry on a FACSCalibur system equipped with a 488 nm Ar-ion laser using the CellQuest software (Becton Dickinson, Franklin Lakes, NJ, USA) and the FL-1 (530/30) filter on at least 2 × 104 events per sample.

### 4.7. Small Extracellular Vesicles (Small EV) Isolation and Characerization

For sEV isolation, cells were incubated for 72 h. Then, medium was collected and the cells and cellular debris were removed from the medium by subsequent centrifugation at 300× *g* for 10 min and 2000× *g* for 10 min, respectively. The medium was passed through a 0.22 μm-pore-size filter (cellulose acetate membrane, Corning) to remove larger vesicles. The sample was concentrated (Amicon^®^ Ultra, with Ultracel-100 regenerated cellulose membrane; Merck, St. Louis, MO, USA) and ultracentrifuged on 2 mL of 20% sucrose cushion for 135 min, 4 °C at 100,000× *g* (MLA-55; Beckman Coulter) [72]. The pellet was resuspended in PBS and subsequently used for nanoparticle-tracking analysis (NTA), transmission electron microscopy, in radioimmunoprecipitation assay buffer (RIPA) for Western blot, and in lysis buffer (*mir*Vana; ThermoFisher) for RNA extraction studies.

#### 4.7.1. Protein Isolation and Western Blot

Pellets of sEVs and cells were resuspended in RIPA buffer supplemented with 100X Halt™ Protease Inhibitor Cocktail (ThermoFisher) and incubated for 15 min on ice. A protein mixture of cells was centrifuged 15 min at 14,000× *g* and 4 °C. All samples were saved at −20 °C for Western blot analysis. The protein concentration was measured using Pierce BCA Protein Assay Kit (ThermoFisher) as described in the manufacturer’s protocol. Approximately 5 μg of sEVs lysate proteins and 10 μg of cell lysate proteins were separated on 4–12% NuPAGE Bis-Tris gel (ThermoFisher) which was running for 70 min at 200 V. Proteins were then transferred to a polyvinylidene fluoride membrane. The transfer was running 90 min at 100 V. Afterwards, the membrane was blocked for 1 h in 5%/PBS milk with shaking at room temperature, and then incubated overnight at 4 °C with the following primary antibodies: rabbit anti-flotillin-1 (1:1000; Cell Signalling), mouse anti-HSP70 (1:200; Santa Cruz, Dallas, TX, USA), mouse anti-CD9 (1:200; Santa Cruz, Dallas, TX, USA), and rabbit anti-calnexin (1:2000; Sigma Aldrich). Then, secondary goat anti-rabbit HRP-conjugated antibody (1:7500; Sigma Aldrich) or goat anti-mouse HRP-conjugated antibody (1:4000; Sigma Aldrich) were applied. The membrane was incubated with Super Signal West Pico Chemiluminescent Substrate (ThermoFisher). Chemiluminescence signals were visualized with LAS-4000 CCD camera (Fujifilm; Tokyo, Japan).

#### 4.7.2. Nanoparticle Tracking Analysis

The average size of sEVs was determined by nanoparticle tracking analysis using Nanosight NS300TM (Malvern Panalytical, Malvern, UK) equipped with syringe pump and autosampler. Briefly, pelleted sEVs were resuspended and diluted 1:200 in filtered (0.22 μm-pore size) and sonicated PBS. For each sample, five videos of 60 s were filmed at the camera level 14 and threshold five. Slide shutter was at 1259 and slider gain at 366. The temperature was set at 25 °C. Software NTA 3.3.- Sample Assistant Dev Build 3.3.203 was used for the detection and analysis.

#### 4.7.3. Transmission Electron Microscopy

Negative contrasting was performed according to Théry et al. [73]. The sEVs were fixed in 2% formaldehyde in 0.1 M sodium phosphate buffer. Five μL droplets were applied to formvar/carbon coated EM grids and dried for 20 min. Grids were put on droplets of PBS and immediately transferred to 1% glutaraldehyde for 5 min. After washing with distilled water, grids were put on droplets of uranyl-oxalate solution for 5 min at room temperature and then on 4% uranyl acetate and 2% methyl cellulose for 10 min on ice. Grids were collected on a loop, excess fluid was blotted off by filter paper, and grids were air dried. Grids were analyzed by a transmission electron microscope (Philips CM100, Philips, Amsterdam, Netherlands), operating at 80 kV.

### 4.8. Total RNA Isolation from Cells and sEVs

Total RNA was isolated using the *mir*Vana miRNA Isolation Kit (ThermoFisher). Briefly, cells were washed with PBS and 300 μL of Lysis/Binding solution was added. The sEVs were put directly in 300 μL of Lysis/Binding solution. Next, the samples were vortexed and 30 μL of miRNA homogenate was added, vortexed, and incubated on ice for 10 min. Then, 300 μL of Acid-Phenol:Chloroform was added and vortexed for 30 s. The mixture was centrifuged 5 min at 10,000× *g*. The upper phase was transferred to a new tube and 1.25 volume of 100% ethanol was added. The mixture was transferred to the filter in the collection tube and centrifuged for 15 s at 10,000× *g*. The flow-through was removed and 700 μL of miRNA wash solution 1 was added and the mixture centrifuged 10 s at 10,000× *g*. Flow-through was removed again and 500 μL of wash solution 2/3 added, and the mixture was centrifuged 10 s at 10,000× *g*. Again, the flow-through was removed and 500 μL of wash solution 2/3 added, and the suspension was centrifuged 10 s at 10,000× *g*. After flow-through removal, with 1 min spin-down, the remaining liquid was removed. The filter was transferred to a new collection tube and total RNA was eluted with 50 μL of water, previously warmed at 95 °C, and the solution was centrifuged 30 s at maximal speed. The concentration of RNA was measured using Synergy H4 (BioTek, Winooski, VT, USA) and the RNA purity was determined using the A260/A280 and A260/A230 ratios.

### 4.9. Quantification of Cellular and sEV miRNA, and sEV mRNA

The process of miRNA to cDNA synthesis was as follows. First, to remove possible DNA traces RNA was treated with Dnase (0.1 μL; Roche) in 10× DNAse buffer (0.5 μL; Roche) in a total volume of 5 μL. The reaction mixture was incubated for 15 min at 30 °C and 10 min at 75 °C. Then, miRNA was transcribed to cDNA using the TaqMan Advanced miRNA cDNA Synthesis Kit according to the manufacturer’s manual (ThermoFisher Scientific). The qPCR mixture consisted of 2.25 μL of cDNA, 0.25 μL of probes, and 2.5 μL of TaqMan Fast Advanced Master Mix (ThermoFisher Scientific) in a total 5 μL reaction. The settings were as follows: 20 s at 95 °C; 40 cycles of 1 s 95 °C, 20 s 60 °C; hold 4 °C. qPCR was performed using ViiA7 (Applied Biosystems, Life Technologies).

In the case of mRNA, RNA was treated with DNAse (Roche) 15 min at 30 °C and then 10 min at 75 °C. Then, RNA was converted to cDNA with the High Capacity cDNA Reverse Transcription Kit (ThermoFisher) together with RNAse Inhibitor (ThermoFisher) with the following settings: 10 min at 25 °C, 120 min at 37 °C, 5 min at 85 °C. qPCR was performed using ViiA7 (Applied Biosystems, Life Technologies, Waltham, MA, USA). A total reaction volume of 5 μL consisted of 2.5 μL TaqMan Fast Advanced Master Mix (Thermo Fisher), 0.25 of TaqMan Probe (ThermoFisher), and 2.25 μL of cDNA. The settings were as follows: 20 s at 95 °C; 45 cycles of 1 s 95 °C, 20 s 60 °C; hold 4 °C. The experiment was performed with three biological replicates, and each biological replicate was tested in triplicate. The reference genes for miRNA were miR-99a-5p [69] and for mRNA were GAPDH [74], beta-actin [75], and 18s rRNA [76]. All probes are listed in Table 7.

### 4.10. Statistical Analysis

The analysis of the relative miRNA and mRNA expression was determined as previously described [77]. Briefly, the expression of each of the study genes was normalized to the expression of the reference genes. Statistical significance was determined by a one-way ANOVA for data with a Gaussian distribution and Kruskal–Wallis for data without a Gaussian distribution in statistical tests.

## 5. Conclusions

GBM is a very complex disease and one of the deadliest types of cancer. Due to the extremely low survival rate, new biomarkers are very much needed that could improve the diagnosis and prognosis and determine the selection of appropriate therapy. The detection of specific RNAs in sEVs could significantly assist in our understanding of extracellular tumor communication and serve as potential and promising biomarkers. In our study, we showed that a higher expression of miR-9-5p and miR-124-3p was related to the sEVs of NCH421k or NCH644 GBM stem cell lines, respectively. Moreover, miR-9-5p was associated with a decreased survival of GBM patients carrying an *IDH* mutation. miR-21-5p expression could differentiate between GBM and lower-grade glioma and between GBM and reference brain, while miR-124-3p and miR-138-5p presented with a lower expression in GBM with respect to the reference brain tissue. Both miRNAs, miR-124-3p and miR-138-5p, target *VIM* gene, which was overexpressed in the U251 and U87 glioblastoma cell lines-derived sEVs. We therefore suggest that the *VIM* mRNA present in sEVs is a specific marker of more differentiated GBM cells compared to stem cells and may serve as a potential biomarker for the characterization of GBM in the body fluids of patients and/or as potential future targets in GBM therapy. Furthermore, miR-9-5p and miR-124-3p may be further explored as potential GBM stem cell biomarkers present in sEVs. miR-9-5p could also be investigated as a prognostic biomarker, indicating the worse survival of glioblastoma patients with an *IDH* mutation. The further validation of candidate RNAs requires the analysis of a larger cohort of samples and finally, in addition, the substantial validation of the cargo in sEVs from different body fluids (blood, cerebrospinal fluid) of patients with GBM. Finally, in this study, only a few miRNAs and according target genes were included and in order to better characterize glioblastoma sEVs, other novel miRNAs or other RNAs could be investigated, for example, by transcriptome sequencing.

## Figures and Tables

**Table 1:** Candidate miRNAs with the corresponding target mRNA determined by the TargetScan, miRDB, and microT-CDS datasets.

miRNA	Gene	TargetScan (Aggregate Pct)	miRDB (Target Score)	microT-CDS (miTG Score)
hsa-miR-9-5p	*FREM2*	0.96	90	0.99
	*NAP1L1*	0.91	74	0.92
hsa-miR-21-5p	*SPRY1*	0.70	95	0.96
hsa-miR-124-3p	*VIM*	0.86	91	0.74
	*NAP1L1*	0.92	61	0.82
	*SPRY1*	0.87	X	0.72
hsa-miR-138-5p	*VIM*	0.70	94	X
	*NCL*	X	X	0.80
hsa-miR-1-3p	*NCL*	0.98	90	1

The prediction probability of miRNA–mRNA pair formation was determined by aggregate Pct (TargetScan), target score (miRDB), and miTG score (microT-CDS). Pct—probability of preferentially conserved targeting (ranging from 0 to 1) [27]. miTG—target prediction score. X—no results obtained.

**Table 2:** Pathway analysis of common genes analyzed by WebGestalt (KEGG, Panther, and Reactome databases).

		Description	FDR			Description	FDR
**miR-9-5p**	KEGG	Focal adhesion	0.021	**miR-124-3p**	KEGG	Apelin signaling pathway	0.012
		**Prostate cancer**	0.021			Cellular senescence	0.012
		ErbB signaling pathway	0.021			Axon guidance	0.012
		Cellular senescence	0.021			**Pathways in cancer**	0.012
		Regulation of actin cytoskeleton	0.021		Panther	x	
		**MicroRNAs in cancer**	0.021		Reactome	Receptor-type tyrosine-protein phosphatases	0.031
		Neurotrophin signaling pathway	0.021			NOTCH2 Activation and Transmission of Signal to the Nucleus	0.031
		**Hepatocellular carcinoma**	0.021			Semaphorin interactions	0.031
	Panther	Ras Pathway	0.009			Signaling by Receptor Tyrosine Kinases	0.031
	Reactome	X				Signaling by MET	0.031
**MiR-21-5p**	KEGG	**Proteoglycans in cancer**	0.019	**MiR-138-5p**	KEGG	X	
		Ras signaling pathway	0.032		Panther	X	
		**MicroRNAs in cancer**	0.038		Reactome	X	
		Neurotrophin signaling pathway	0.043	**MiR-1-3p**	KEGG	**MicroRNAs in cancer**	0.000
		EGFR tyrosine kinase inhibitor resistance	0.043			Adherens junction	0.010
	Panther	X				Bacterial invasion of epithelial cells	0.010
	Reactome	Signaling by NTRK2 (TRKB)	2.62 × 10^−3^			Axon guidance	0.029
		Signaling by NTRKs	2.62 × 10^−3^			**Proteoglycans in cancer**	0.029
		Signaling by BMP	2.69 × 10^−3^			EGFR tyrosine kinase inhibitor resistance	0.035
		Signaling by Receptor Tyrosine Kinases	4.94 × 10^−3^			**Choline metabolism in cancer**	0.035
		MET activates PI3K/AKT signaling	4.94 × 10^−3^			Regulation of actin cytoskeleton	0.035
		PI-3K cascade:FGFR3	5.14 × 10^−3^			Hepatitis B	0.035
		PI-3K cascade:FGFR4	5.97 × 10^−3^			Cocaine addiction	0.042
		Signaling by FGFR3	5.97 × 10^−3^			**Viral carcinogenesis**	0.042
		Signaling by FGFR4	5.97 × 10^−3^			**Renal cell carcinoma**	0.042
		Signaling by EGFR	5.97 × 10^−3^			Hippo signaling pathway	0.042
		Signaling by FGFR3 in disease	5.97 × 10^−3^			Spliceosome	0.043
		Signaling by FGFR3 point mutants in cancer	5.97 × 10^−3^			Human papillomavirus infection	0.044
		PI-3K cascade:FGFR2	6.63 × 10^−3^			**Thyroid cancer**	0.044
		Downstream signaling of activated FGFR3	8.67 × 10^−3^			Cellular senescence	0.044
		Signaling by FGFR3 fusions in cancer	9.29 × 10^−3^			Pathogenic Escherichia coli infection	0.044
		Signaling by FGFR1	9.29 × 10^−3^		Panther	X	
		Downstream signaling of activated FGFR4	9.77 × 10^−3^		Reactome	Signaling by Receptor Tyrosine Kinases	0.000
		Signaling by FGFR4 in disease	0.010			Clathrin-mediated endocytosis	0.010
		GAB1 signalosome	0.010			RHO GTPases Activate WASPs and WAVEs	0.010
		PIP3 activates AKT signaling	0.010			Signaling by VEGF	0.013
		Downstream signaling of activated FGFR2	0.012			RNA Polymerase II Transcription	0.028
		Signaling by FGFR in disease	0.020			VEGFA-VEGFR2 Pathway	0.028
		Intracellular signaling by second messengers	0.022			Gene expression (Transcription)	0.035
		MAPK family signaling cascades	0.022			Signaling by RAS mutants	0.035
		Signaling by TGF-beta family members	0.022			Diseases of signal transduction	0.035
		Signaling by FGFR1 in disease	0.025			EPH-Ephrin signaling	0.041
		Interleukin-4 and Interleukin-13 signaling	0.028			Generic Transcription Pathway	0.041
		**Constitutive Signaling by Aberrant PI3K in Cancer**	0.028			Platelet activation, signaling and aggregation	0.042
		Signaling by FGFR2	0.031			RHO GTPase Effectors	0.042
		Signaling by FGFR2 in disease	0.034			EPHB-mediated forward signaling	0.042
		Diseases of signal transduction	0.034			Hemostasis	0.045
		Generic Transcription Pathway	0.035			Regulation of actin dynamics for phagocytic cup formation	0.048
		PI3K Cascade	0.035			Membrane Trafficking	0.049
		Signaling by NODAL	0.038				
		Gene expression (Transcription)	0.038				
		MET activates PTPN11	0.039				
		PI-3K cascade:FGFR1	0.040				
		IRS-mediated signalling	0.042				

Pathways directly involved in cancer are marked in bold. Each miRNA section is separated by color. X—no results obtained. KEKEGG, Pa—Panther, ReReactome.

**Table 3:** Statistical analysis of the qPCR results of miRNA expression in GBM, LGG, and reference brain tissue samples.

miRNA	Tissues	Significance	*p*-Value
miR-9-5p		ns	
miR-21-5p	GBM vs. LGG	*	0.0146
	GBM vs. reference brain tissue	****	<0.0001
miR-124-3p	GBM vs. reference brain tissue	*	0.0178
	GBM vs. LGG	*	0.0278
miR-138-5p	GBM vs. reference brain tissue	*	0.0143
miR-1-3p		ns	

* *p* < 0.05, **** *p* < 0.0001, ns – non significant. GBM—glioblastoma; LGG—lower-grade glioma.

**Table 4:** Statistical analysis of the qPCR results of the miRNA expression in sEVs and originating cells.

miRNA	sEV			Cell		
miR-9-5p	U251 vs. NCH421k	**	0.0048	U251 vs. U87	**	0.0028
	U87 vs. NCH644	*	0.0362	U251 vs. NCH421k	****	<0.0001
	U87 vs. NCH421k	****	<0.0001	U87 vs. NCH644	*	0.0284
	NCH644 vs. NCH421k	****	<0.0001	U87 vs. NCH421k	****	<0.0001
	NCH421k vs. astrocytes	****	<0.0001	NCH644 vs. NCH421k	****	<0.0001
				NCH421k vs. astrocytes	****	<0.0001
miR-21-5p	ns			NCH644 vs. astrocytes	**	0.0052
				NCH421k vs. astrocytes	**	0.0042
miR-124-3p	U251 vs. NCH644	****	<0.0001	U251 vs. NCH644	****	<0.0001
	U87 vs. NCH644	****	<0.0001	U251 vs. NCH421k	**	0.0092
	NCH644 vs. NCH421k	***	0.0002	U87 vs. NCH644	**	0.0028
	NCH644 vs. astrocytes	****	<0.0001	NCH644 vs. astrocytes	****	<0.0001
				NCH421k vs. astrocytes	**	0.0075
miR-138-5p	U251 vs. U87	*	0.0301	U251 vs. U87	****	<0.0001
				U87 vs. NCH644	****	<0.0001
				U87 vs. NCH421k	****	<0.0001
				U87 vs. astrocytes	****	<0.0001
miR-1-3p	ns			U87 vs. NCH421k	***	0.0007
				U251 vs. U87	*	0.013
				NCH644 vs. NCH421k	*	0.0242
				NCH421k vs. astrocytes	*	0.0140

* *p* < 0.05, ** *p* < 0.01, *** *p* < 0.001, and **** *p* < 0.0001; ns—non-significant.

**Table 5:** Statistical analysis of the qPCR results of the mRNA expression in sEVs.

Gene	Cells	Significance	*p*-Value
*NAP1L1*	U251 vs. U87	*	0.0466
	U251 vs. astrocytes	***	0.0002
	NCH644 vs. astrocytes	*	0.0300
*VIM*	U251 vs. NCH644	****	<0.0001
	U251 vs. astrocytes	****	<0.0001
	U87 vs. NCH644	***	0.0003
	U87 vs. astrocytes	****	<0.0001
*NCL*	NCH644 vs. U87	*	0.0418

* *p* < 0.05, *** *p* < 0.001, and **** *p* < 0.0001. The results for *FREM2* and *SPRY1* genes are not presented because there were no statistical differences observed.

**Table 6:** Summary of significant results. Arrows indicate the upregulation or downregulation of specific RNA.

miRNA or mRNA	sEVs	Cell Lines	Tissue
miR-9-5p miRNA	↑ NCH421k	↑ NCH421k	↑ associated with the poor survival of IDH mutant GBM patients ↓ associated with the better survival of GBM patients (regardless of IDH mutation)
miR-21-5p miRNA	/	↓ NCH421k, NCH644	↑ GBM vs. LGG, reference tissue
miR-124-3p miRNA	↑ NCH644	↑ NCH421k, NCH644	↓ GBM vs. LGG, reference tissue
miR-138-5p miRNA	/	/	↓ GBM vs. reference tissue ↑ associated with the poor survival of IDH mutant GBM patients
*VIM* mRNA	↑ U251, U87	/	

IDH—isocitrate dehydrogenase; GBM—glioblastoma; LGG—lower-grade glioma. ↑—higher expression, ↓—lower expression.

**Table 7:** List of the TaqMan probes used in the experiments. All the probes are from ThermoFisher.

miRNA	Assay
miR-9-5p	478214_mir
miR-21-5p	477975_mir
miR-124-3p	480901_mir
miR-138-5p	477905_mir
miR-1-3p	477820_mir
miR-99a-5p	478519_mir
SPRY1	Hs00398096_m1
vimentin	Hs00958111_m1
FREM2	Hs01388268_m1
NAP1L1	Hs01590181_g1
nucleolin	Hs01066668_m1
GAPDH	Hs99999905_m1
18S	Hs99999901_s1
Beta-actin	Hs99999903_m1

## Supplementary Materials

Supplementary materials can be found at https://www.mdpi.com/1422-0067/21/22/8491/s1.

## Abbreviations

EV	Extracellular vesicle
FDR	False-discovery rate
FREM2	FRAS1-related extracellular matrix protein 2
GBM	Glioblastoma
IDH	Isocitrate dehydrogenase
LGG	Lower-grade glioma
MGMT	O-6-methylguanine-DNA-methyltransferase
MRI	Magnetic-resonance imaging
NAP1L1	Nucleosome assembly protein 1-like 1
NCL	Nucleolin
NTA	Nanoparticle-tracking analysis
sEV	Small extracellular vesicle
SPRY1	Protein sprouty homolog 1
VIM	Vimentin
