# Supplementary material for: Analysis of miR-9-5p, miR-124-3p, miR-21-5p, miR-138-5p, and miR-1-3p in Glioblastoma Cell Lines and Extracellular Vesicles
Source: Int J Mol Sci. 2020 Nov 11;21(22):8491. doi: 10.3390/ijms21228491 (PMC7698225; doi:10.3390/ijms21228491)
Supplement: Supplementary file 1 [file ijms-21-08491-s001.pdf]

International Journal of *Molecular Sciences*

MDPI

Supplementary materials

Analysis of miR-9-5p, miR-124-3p, miR-21-5p, miR-138-5p, and miR-1-3p in Glioblastoma Cell Lines and Extracellular Vesicles

Alja Zottel [1,*], Neja Šamec [1], Ana Kump [1,2], Lucija Raspor Dall'Olio [1], Pia Pužar Dominkuš [1], Rok Romih [3], Samo Hudoklin [3], Jernej Mlakar [4], Daniil Nikitin [5,6], Maxim Sorokin [6,7,8], Anton Buzdin [5,7,8,9], Ivana Jovčevska [1,†] and Radovan Komel [1,*,†]

1 Medical Centre for Molecular Biology, Institute of Biochemistry, Faculty of Medicine, University of Ljubljana, 1000 Ljubljana, Slovenia; neja.samec@mf.uni-lj.si (N.Š.); ana.kump16@gmail.com (A.K.); lucijaraspor@gmail.com (L.R.D.); pia.puzar-dominkus@mf.uni-lj.si (P.P.D.); ivana.jovcevska@mf.uni-lj.si (I.J.)
2 Jožef Stefan International Postgraduate School, 1000 Ljubljana, Slovenia
3 Institute of Cell Biology, Faculty of Medicine, University of Ljubljana, 1000 Ljubljana, Slovenia; rok.romih@mf.uni-lj.si (R.R.); samo.hudoklin@mf.uni-lj.si (S.H.)
4 Institute of Pathology, Faculty of Medicine, University of Ljubljana, 1000 Ljubljana, Slovenia; jernej.mlakar@mf.uni-lj.si
5 Shemyakin-Ovchinnikov Institute of Bioorganic Chemistry, Russian Academy of Sciences, 117997 Moscow, Russia; nikitin@oncobox.com (D.N.); buzdin@oncobox.com (A.B.)
6 Oncobox ltd., 121205 Moscow, Russia; sorokin@oncobox.com
7 Laboratory of Clinical and Genomic Bioinformatics, I. M. Sechenov First Moscow State Medical University, 119146 Moscow, Russia
8 Moscow Institute of Physics and Technology (National Research University), 141700 Moscow region, Russia
9 OmicsWay Corp., Walnut, CA 91789, USA
* Correspondence: alja.zottel@mf.uni-lj.si (A.Z.); radovan.komel@mf.uni-lj.si (R.K.); Tel.: +386 1 543 7662
† These authors share lead authorship.

Received: 31 May 2020; Accepted: 6 November 2020; Published: date

Supplementary Table 1. The list of common genes between datasets.

	Target genes
MiR-9-5p	ONECUT2, YBX3, LYVE1, POU2F1, SLC50A1, TRPM7, AP1S2, PDK4, LDLRAP1, KCNJ2, SGMS2, MTHFD2, FRMD6, FSTL1, RIC3, LIN28B, PRDM6, RAB34, SLC31A2, ANK2, SFXN2, CLDN14, C2orf88, CXCL11, SOCS5, VAV3, RANBP17, MAGT1, CCNDBP1, GALNT3, GABRB2, CAPZA1, TNC, SLC10A3, TESK2, POU2F2, CCNG1, IPO4, SCRIB, TNFAIP8, RNF150, NID2, NXPE3, CALB2, FOXN2, MDGA2, KLF5, LEP, DYRK1B, MAEA, PCGF6, ENPEP, SMARCD2, EMB, ARMCX2, MYOCD, UBE3C, FYTTD1, YPEL2, MTMR2, SHC2, PIRT, FOXP4, MYH1, TRIM71, CMTR2, AP3B1, DOK6, C21orf91, ANO1, CCDC43, FAM19A5, KCNMB2, PRDM1, PI4K2A, LURAP1L, EIF4E3, TOMM20, DLX3, SEC23IP, NTNG1, SNX7, PRRX1, WASF2, ANP32B, NR5A2, GPR137C, ITGB4, CPEB2, ARID3B, GDNF, TMEM109, TBPL1, SUDS3, CCAR2, GCH1, GOT1, CCDC138, SIRT1, MAP3K2, OGDHL, GRHL1, RASSF3, DIXDC1, C1QL1, ALCAM, SPECC1L, VRTN, ALAD, M6PR, IGFBP3, PTAR1, PRPS2, EHD4, ITM2C, PALMD, DNAJC14, ZC3H12A, KCTD12, PCSK2, NAP1L1, CDK8, ZKSCAN1, DDHD2, COL15A1, RAB27B, DSE, INHBB, PAK4, IGF2BP2, SHC1, CSGALNACT1, SLC8A1, CHST15, ATL2, COL27A1, RHOJ, PANK3,

	CSNK1A1, IGF2BP3, ULK2, GMEB2, NEDD4, ZBED3, TRERF1, FAM13C, MAN1A2, TCF7, PBRM1, DIAPH2, CA7, P4HA2, BAG4, ENTPD5, STMN1, HLTF, VANGL1, ZNF354A, FGF5, POU2F3, LZTS2, LIN28A, RAB8A, UBE2Z, PARG, TMEM87B, KCNK4, CRYBG3, GZF1, FAM19A4, UHMK1, SMAP2, KLHL1, COL9A1, ITPRIPL2, GJA3, TGFBR2, CNTFR, HOXA11, MYO1C, TSC22D2, SRSF10, SYAP1, RNF111, SLC25A24, FREM2, ARHGAP24, ANKRD13A, ZBTB39, RNF19A, FBXL2, ERG, MAP3K3, SLC39A9, ESYT1, MYPN, OTUD3, ARID1A, RNF128, CCNE2, MAP2K7, DHX40, OTUD7B, CPEB4, ELOVL4, KLHL42, FAM117A, ZBTB41, VAT1, CRIM1, SHISA2, CCSER2, TTYH2, ZIC5, RNF24, AP4E1, PXDN, HUNK, KLHL18, LDLRAD3, ITPKC, VGLL4, FAM199X, EFEMP1, UBASH3B, SLC26A2, CLCA2, STAM, DNAJC3, TBC1D8, UTRN, NPY2R, C18orf25, BCL2L11, SLC9A1, NHLH2, TBC1D4, TES, COG3, FOXG1, MBNL1, ZC3H10, SLC39A14, GSKIP, HK2, ARHGEF2, EIF5A2, KIAA2013, BAHD1, ZNF365, STEAP3, ASB7, PPM1F, NID1, ATOH8, LPP, FAF2, NHSL1, SIK1, ZNF395, TLK1, TBC1D22A, PIK3R3, PLBD2, ADAMTS3, CLOCK, PEX5L, TMEM170B, CPEB3, FBXW2, DPF3, MRFAP1, KIF13A, SCYL3, RBMS3, EN1, SLC18A2, ITIH6, RYBP, MDGA1, BCL6, PTCHD1, ALPL, CNOT7, FBXL3, MMP16, PPP6R3, CNOT6L, CXCR4, LZTS3, RPP14, MARCH6, FGF9, PPIP5K1, PIGZ, AEBP2, IPO13, COLEC12, MIER3, SLC6A6, HSP90AA1, NUTF2, HLCS, PTMA, SH2B3, TAF4B, CMTM6, PYGO2, STAC, STK38L, ANXA2, BTBD10, NCOA7, PHTF2, REEP3, SPTSSA, SLC5A3, VAMP3, CNNM2, CREBRF, CNTN4, SERINC5, TSNAX, KIF1C, DRD2, MAP3K1, PIGM, EPHB4, HES1, PHF8, BEND4, NR2E1, EDEM3, SLAIN2, CAMKK2, RAP1B, SDC2, SH3PXD2B, SRSF1, STT3B, PIK3CB, ARHGEF17, C16orf70, IFFO2, DBNL, MLLT3, AR, C5orf24, TRAM1, AMBRA1, SLC19A2, AMMECR1, CHMP2B, ELMOD2, S100PBP, STARD13, ARL4C, FOXP1, BNC2, GREB1, SMOC2, WIZ, DGKH, AP1S3, RBMS1, CEP104, MAP1A, ATF2, OPCML, EN2, SGSM1, HS3ST3B1, ST8SIA4, ADPGK, BICC1, SACS, PHIP, SORT1, UBE2Q1, NMT2, CC2D1B, TULP4, ENTPD1, MTHFD1L, EFNA1, RASGRF2, COL18A1, SNRK, CUL4A, UBE2H, SLC25A36, NMT1, MME, LARP1, PHF20L1, SPTLC2, ARID1B, TMEM248, NEK1, SLC9A7, BACE1, TNFRSF21, ADAMTS5, SDC1, PPP2R2A, MBTPS1, FAM8A1, KIAA1217, WDTC1, GAD1, SHROOM3, MYH9, RBM24, GOPC, HIPK1, GSK3B, GPBP1L1, SLC27A4, TMTC1, ADCY5, SBNO1, KIAA1549, CNNM1, ETS1, SOS1, FAM155B, SNAP23, SPTSSB, RALGDS, RNF169, CBLN4, CCDC126, SIX4, LSM14A, PRRT3, ITGA6, FXR1, RUNDC3B, DGCR8, FBRS, CPEB1, ARFGEF2, DIO2, MYLK, ARFGEF1, RCOR1, LMX1A, GPRASP2, IKZF5, OXSR1, SYT4, ZBTB34, MAP1B, PLEKHA1, TXNDC5, GRIK3, FAM118A, TSPAN9, PAK6, EPHA7, RC3H1, CBFA2T2, PEAK1, PPIP5K2, ELAVL1, SYNJ1, ARNTL2, ATXN3, DIAPH1, AJUBA, HIPK3, FAM117B, RHOBTB1, ZNF236, FAM43B, SYNJ2BP, SRCAP, PEG3, BEND3, FBXL16, FRYL, RSPRY1, NFIC, GCLM, ANKH, AATK, KDM5A, ATP8B2, CC2D1A, CBX5, KPNB1, PHF13, SHROOM4, ZNF367, FRMD4A, RORA, ATP7A, VDAC3, WARS2, ASXL3, NCOA3, MAPKAPK2, HECTD1, KIAA0930, FBN2, INSIG1, DENND3, SLC30A5, KLHDC10, UHRF1BP1, CCNT1, PPARA, FMR1, SRF, NCOR2, FSD1L, HIPK2, ZNF362, SLC1A1, SAMD8, SPAG9, VCAN, PTBP3, C5orf30, SMARCE1, CHSY1, UNC80, PAX5, PCDH7, RANBP2, FYCO1, ANKRD52, UBR5, PTEN, ZFHX4, FOXO1, EIF5, SNTB2, UBFD1, BAZ2B, NFATC3, KIF13B, SHB, KCNQ3, ID4, LIFR, PCGF5, SSX2IP, TRAF3, ARCN1, ADAM10, CREB5, ANKRD12, GPC6, AMER1, UBN1, CLCN4, VCL, LMBRD2, FLRT3, PLAG1, TNS1, TENM1, PRLR, CHMP1B, PCDH10, MGA, CTDSP2, EVI5, PSD3, PHLDB1, FAM126B, ADCY9, SZRD1, UACA, FOXRED2, SHROOM2, PITPNM2, ABL2, UNC13A, TMEM164, NFASC, CDC73, AMOTL2, ESRRB, CCDC50, MTF2, KCNA1, CCDC6, CLCN5, ICMT, AMOTL1, GPATCH8, LIN7A, ATP1B1, CCDC88A, ZBTB44, PAK3, PKIB, SYT1, RAB5B, UNC5D, CEP170B, ATG14,

	ATP11A, CNTN3, SLC14A1, NCOA2, UBE4B, HIC2, ELAVL4, CEP350, ZNF704, CCNT2, MLXIP, SCUBE3, PACSIN1, SLC4A1, HDAC5, ZDHHC21, ZBTB14, ANKRD29, SRGAP3, NRP1, GUCY1A2, ATP11B, SLC20A2, CDH11, NEO1, HOXB13, TNRC6A, TNRC6B, PTGFRN, SCUBE2, ZEB2, REST, CACNB2, MEF2C, FOXN3, ILDR2, RMND5A, TRIM66, AFF1, PLEKHA6, RAPH1, ARL1, PDCD6IP, GEMIN5, FRMPD4, PITPNC1, MCMBP, RERE, ASXL1, TENM4, MARCKS, ITM2B, FKBP7, FAM107B, UNKL, SLC44A1, SAR1B, TRIM55
miR-21-5p	KRIT1, IL12A, FASLG, FGF18, CCL1, PLEKHA1, RSAD2, YOD1, PELI1, TGFBI, ARMCX1, MATN2, SKP2, NTF3, TIMP3, SMAD7, SATB1, FAM13A, RP2, RTN4, ARHGAP24, UBE2D3, LRRC57, DUSP8, PDCD4, SOX5, RMND5A, OLR1, SPRY1, S100A10, RALGPS2, GLIS2, KLF5, SPRY2, ELF2, RECK, PCBP1, SLC16A10, ST3GAL6, KBTBD6, PPP1R3A, SKI, STAG2, CD69, CPEB3, PCSK6, SESN1, BCL7A, GLCCI1, GABRB2, LANCL1, ST6GAL1, ASF1A, PBRM1, JAG1, CHIC1, TMEM170A, DCAF7, HIPK3, TIAM1, MAP3K1, CASKIN1, RBPJ, NFIB, PLAG1, ACVR1C, THRB, CSRNP3, STAT3, ZSWIM6, RASA2, PAG1, KLHL42, ADNP, NFIA, PIK3R1, ZNF704, FRS2, VASH2, NIPAL1, LIFR, KLF6, CADM2, GATAD2B, FGD4, MSX1, SMARCD1, MIA3, CBX4, TAGAP, FBXO28, EPHA4, NEGR1, VCL, KLHL15, BTG2, TESK2, COL4A1, CDC25A, AKIRIN1, CCL20, PIKFYVE, ANKRD46, TET1, PDZD2, AGO4, IL6R, GPC4, HGF, ALX4, BMPR2, MTMR12, KLF3, RASGRP1, SCML2, BNC2, SOX7, SLC30A10, GAB1, CREBRF, PURB, CNOT6, MEF2C, RBMS3, SUZ12, EHD1, ACVR2A, MPRIP, PAIP2B, BCL11B, STRN, UNC80, TNRC6B, HNRNPU, KLF12, GID4, PRPF4B, YAP1, GTPBP10, RAB11A, NFAT5
MiR-124-3p	RHOG, CTDSP1, SNAI2, LRRC58, B4GALT1, SLC10A7, VAMP3, MAGT1, SERINC2, SERP1, TRIM45, ITGB1, CEBPA, SPOPL, RAB27A, CHIC1, PTBP1, SLITRK6, PTPN12, PHF19, RNPEPL1, PPFIBP2, LPP, RFX4, PTBP2, FLOT2, TUB, TMEM134, RAB34, CPT1A, CNEP1R1, TBX22, SIX4, C11orf87, SLC50A1, VPS37C, NFATC1, ASPA, GXYLT1, BMP6, SLC31A2, CD164, TARBP1, PIK3C2A, PPP1R13L, MAP3K2, DHRS1, TTL, MITF, PI4K2B, PTPN9, LRRC1, SLC16A1, FZD4, IQGAP1, CBX2, SLC26A2, FOXQ1, KIAA1671, VAT1, CREBRF, GNG10, IL11, POLR3G, ECI2, TMED1, FRMD8, STT3A, ATP6V0E1, EDEM1, EYA4, CBL, PCDH8, BLOC1S6, CTSH, CHP1, LMAN2L, PARP16, SLC30A2, SLC16A13, ALG2, CTNND1, RAD17, MAP2K4, PGRMC2, SNTB2, RAB3D, KCNK10, RARG, PTBP3, PTTG1IP, TBX19, FRMD4B, TOR3A, TMEM109, TCTA, SDF2L1, ELK3, MDK, SMAD5, MYZAP, FAM129B, CXADR, MIB1, KLHL24, GPT2, EVI5, WASF2, DNAJC25-GNG10, VIM, LYSMD3, APLN, ZKSCAN8, SCAMP2, CTDSP2, THAP2, TSPAN15, C3orf38, HRCT1, RBMS1, RFFL, SLC30A7, SFT2D2, TFEB, PROX1, SMOX, MAPK14, FPGS, QSER1, PIM1, C4orf46, SBNO2, KCNK2, EIF3B, GGA2, STOM, CC2D1B, EPHX4, ATP7A, PECR, OVOL2, CYB5A, EML6, LHX2, ASCC2, PLP2, RWDD4, QKI, LAMC1, TMCO3, IAPP, SHPK, AP1M2, RBM33, APEX2, RASSF5, SERTAD2, EPS8, UNC119B, KANK1, CD151, PGM1, VPS35, RELA, DDX3X, MSRA, ZNF449, RAB11FIP1, PNPLA2, G3BP1, DMRTA1, PGF, APH1B, SLC35F5, CTDSPL, ZKSCAN4, GATA6, NECAP2, PRRX1, SNX18, PLEKHF2, SLC17A5, CERS2, MARVELD1, CASQ2, RNF128, LRRC57, C2orf88, SOX8, RRAS, KIF13B, CRYBG3, SPPL2A, RYK, FAM133A, SH3PXD2A, MCUR1, DLX5, SERTAD4, DDX6, NFIB, ACAA2, SORD, F11R, USP2, FLOT1, FAR1, CHSY1, MANBAL, LITAF, CTNS, CRTC3, SLC7A2, HECTD2, SRSF6, GPAM, PRPS1, PAQR8, AMMECR1, SNTA1, TEAD1, GMCL1, COL4A1, SLC31A1, IMPACT, MYRF, OGFOD3, SMCO4, ATP1A1, NIPA1, TRMT10A, PQLC3, GCDH, NID1, ZCCHC24, EYA2, ELOVL5, SPRY3, DENND6A, LMF2, SLC15A4, KIAA1958, CASC3, CLEC1A, ATMIN, CDH2, GCH1, CHIC2, ARFIP1, DHCR24, AKT1S1, C2orf69, RNF125, RHBDF1, SAMD12, CMPK1, HADHA, SLITRK4, CAV1, SYT14, P4HA1, LMO4, NR3C1, E2F5, LCLAT1, AHR, ZMPSTE24,

	FAM76B, XPO4, TMEM87B, NAP1L1, PLEKHH1, STK36, WIPF2, KLF4, ZNF219, GRB2, RAVER1, EGR2, FXR1, PAPSS2, PALLD, PHF6, TRIB3, TPD52L2, TTC26, CAPN6, TRAF6, PTPRZ1, WDR44, FAM199X, KIF2A, PRKAG2, NKAP, USP1, PAM, RYR3, LIMD2, NEGR1, GLIS2, PDE4B, CREB3L2, FAM171A1, PKN2, CLOCK, NRG1, GALNT9, SOS2, ZKSCAN3, PAPOLG, MYH10, IRF2BPL, VCAN, DLX2, CDCP1, EGR1, PLXNA3, HIPK3, SLC7A14, HIVEP2, FCHO2, MSRB1, RCOR1, RNF144A, SFT2D3, CPNE3, SCN4B, SUCO, MTMR6, AK2, SMARCC1, HIVEP3, PARP9, ANXA5, BCL2L11, PLXNB2, CAPN1, CPEB1, RAP2B, INSIG2, ZNF608, CCND2, OSBPL3, DACT1, RHOU, TEX261, KLLN, FSD1L, ZMAT3, FGFR1OP, ITGA7, LRCH4, TRIM39, KAT7, SLCO4C1, PIP4K2C, SLC9A9, SP1, RPIA, LEMD3, NEURL1B, POGLUT1, NFIX, LIPE, SNX30, RRAGD, PHACTR2, MYCBP, RNF141, SLC35A4, FMR1, SHC4, BCL11B, BCL6, JAG1, CHODL, SVIL, SLC25A30, HEATR1, YEATS2, SNX6, FUT10, USO1, LNX2, DSC1, GFPT2, AIDA, MDGA2, NRP1, MYO10, GRIA2, FAM174B, SURF4, PPM1F, SLITRK3, MYH9, NYNRIN, ARHGEF37, TOM1, ETS1, KIF3A, CDH4, SLC9A2, CPD, SH3BP5L, RANBP10, CNN3, GPD2, NR3C2, ITPR3, ANTXR2, ZFP36L2, JAM2, ROCK1, RBL1, MCF2, MEF2A, TMEM184B, ARL10, MYO5B, NFIA, MYO1E, TWSG1, SPECC1L, CDH9, PUS7, SPOCK3, CACUL1, SLC39A9, KIAA2013, ITPKB, TCF3, NXT2, NAP1L5, POC1B, VPS4B, NRP2, TPST2, IPO8, MYADM, C1GALT1, SH2B3, MYH11, PML, CALCOCO1, PPP1R3B, SREK1, ADAM19, SLCO5A1, SRGAP1, MOB1B, CDK4, SERTAD3, SNCAIP, PLEC, NFIC, RFX1, KLF6, AMOTL1, PITPNA, CLMP, SLITRK2, ADNP2, CGN, GIT2, GLI3, XYLT1, USP14, HDAC4, TLN1, PLCB1, AIF1L, IFFO2, RBM24, MTM1, SIGMAR1, LRFN1, RREB1, ANXA7, FXR2, RAB22A, GZF1, STK38, FSTL5, G3BP2, REEP1, KIF16B, FAM53B, TAOK1, TRAM2, EDNRB, AMMECR1L, RRBP1, MYRIP, PRKG1, METAP2, GNAL, NRCAM, RFX3, SEMA6D, FKBP15, E2F3, ATXN1L, RHOQ, PEA15, MYLIP, HIPK1, FAM219B, PLIN3, VPS37B, TBC1D9B, CACNA2D1, ATL3, TOMM34, ARL5B, ATP6V0A2, COTL1, KDELR2, FCHSD2, RNF135, NUP210, AHRR, MTR, BTG2, TRPS1, STRN, UNC5D, GNA13, SCD, MCU, ZNF503, KIAA1324L, SAMD4A, FAM117A, LPCAT3, AP3M1, ECE1, GLRB, NFATC2, MOCS1, STK35, IL17RD, ENPP4, PRKAA2, TET1, RALGDS, RNF216, SLC25A37, NAPEPLD, UBN2, TDRP, INSM1, ARHGDIA, ERN1, PIK3CA, JAG2, CBLN4, DNMT3B, SNAP29, DSG2, ANO5, RNF165, CHD1, KPNA3, HBP1, SLC5A3, DUSP3, MBOAT2, PCYOX1, SLC22A5, CHST1, RALGPS2, CLIC6, FRS2, TNRC6B, NOL4, PRKD1, SUCLG2, RNF213, GPR37, ALDH1L2, ZBTB11, GAS1, ITPRIPL2, VSNL1, RIMBP2, USP48, ULK2, NEK9, LMO3, RAPGEF1, ADCY9, MPP6, PGAP1, CPNE5, PCSK6, BTBD7, ENAH, TP53INP1, KCNQ5, MAPRE1, ZBTB6, ELL2, EFCAB14, SDAD1, RAVER2, SEPSECS, KIF26B, SH3RF1, MPC1, FKBP1B, SLITRK5, INO80D, CLIP1, BCAT1, RGS9, LRIG1, MED26, MARCH7, EYA3, EFHD2, ETV1, TUBG1, STAT3, PARP8, SP3, SCML4, CLDND1, NPTN, CBFB, CEP85L, AFF4, CASC4, OSBP, FRAS1, SLC33A1, FAM78A, FSTL1, RYR2, UHMK1, RASGEF1A, CNTN1, HLF, SH3KBP1, TBC1D13, DEPTOR, LONRF1, ANAPC7, GRIN3A, TACC1, DNASE2, VANGL1, SVIP, GSS, PTAR1, ITGA3, RTCB, PTPRD, MARCH8, RBM12, EZH2, CDYL2, KCNJ12, BAZ2B, CDR2L, SAP30L, ERF, SESTD1, CUL5, NEUROD1, TMEM168, ITPRIP, MAT2A, PMEPA1, TENM1, CELSR1, TMEM41A, BACH2, GLTP, FNBP1L, CD276, LRRFIP2, NHLRC2, LPIN1, ZRANB2, MTX3, RPS6KB1, C2orf68, MLLT3, STK4, XKR6, GALNT13, RAB10, WIPF3, BTBD10, SP2, FZD8, SLC7A8, DIAPH1, PRLR, KCNJ6, MECOM, ROCK2, ELK4, DMXL1, CAMTA2, FBXO30, VAT1L, SUGT1, FAM122B, OTUD1, FAM104A, NLGN4X, DGKH, IMPAD1, EEA1, EPHA3, PCGF5, KATNBL1, CCDC50, THSD7B, RBM20, MAP7, SNIP1, PDE4D, LCP1, UBE3A, DPH3, KLHL28, YIPF6, BRWD1, NACC2, MTDH, PRPF40A, CDK13, CDCA7, RAB2A, SASH1, NKAIN2, AFF1, MED12L, SYNPO2, DAP, RALGPS1, SEMA6C, FRMD4A,

	EN2, GGPS1, HTR2C, APBB2, OXSR1, OSBPL6, LRP6, GLCE, NR5A2, PNN, ACSL1, OTUD4, RALA, NR4A3, ALCAM, BICD2, BRWD3, GPC4, UBE4A, RLIM, TBC1D5, ERMP1, PDZD2, PALM2, IST1, PTPN1, ENTPD5, RBMS3, ANXA11, LIMS1, JAKMIP3, KCNJ2, ZFAND3, NFAT5, HEBP2, OSBPL11, HIF1AN, NAV1, ADIPOR2, PRR14L, MBNL3, LZIC, DGAT2, TOX, TMCC3, BMPR1B, SHANK2, DCTN4, FAM160A1, GDAP1L1, TFDP2, USP30, CLMN, SOS1, ZNF706, MARK1, FAM177A1, C1orf21, BCL2L13, ESYT2, RNF217, FZD5, SYPL1, NAA15, ITGA11, RAPH1, ADCYAP1, MGAT4A, HIPK2, SLC1A4, OSBPL8, MYNN, COL12A1, ARFGEF2, SPOCK2, WDFY3, DYRK2, GNAI1, TOR1AIP2, GDAP2, EFHC1, CACNB4, GRID1, SEMA6A, ANKLE2, SRGAP3, SPTLC2, HDAC5, BACE1, TANC2, CNTN3, DMD, RAB11A, EFNA5, RHBDL3, SH3GLB1, RB1CC1, AGPS, ASF1A, LIMCH1, FARP1, SNX16, SESN3, CEP350, MECP2, NUFIP2, WIPF1, MTF1, ALG9, SPTY2D1, VAMP4, CADM1, CCDC6
miR-138-5p	SYT13, GTPBP1, EIF4EBP1, FOXC1, CLVS1, DUSP16, SLC35F1, DCP1A, RARA, BNIP3L, KLF11, VSTM2L, FOXP4, NKAIN1, RMND5A, ZNF704, JAZF1, CREB3L2, CCND3, PDE3A, AMMECR1, RHOC, RDH8, SH3GL2, SRRM4, NFIB, IGLON5, PPIP5K1, SH2B3, TCF3, CLNS1A, EBPL, NSFL1C, UBP1, ERI1, EID1, CLIP1, SULF2, MANEAL, PLLP, FEM1C, LYPLA1, KDM6B, TIPARP, STOX2, NFIX, SEH1L, PDIK1L, FERMT2, RCAN2, LZTS3, H3F3B, ST6GALNAC4, KBTBD4, PPARD, CHST10, UIMC1, SOX12, ZNF385A, ADCYAP1R1, CALN1, ANKRD54, CLN5, TNFSF4, NSMF, NEUROD1, SLC17A7, DTX4, PDE7B, SOCS5, TMEM198, ZNF444, TMTC4, NPPC, SNCAIP, ST3GAL6, TCF4, PIP4K2C, USP10, AGO1, PRRT4, UPF2, RIMS2, UHRF1BP1, SIRT1, THAP11, THRB, BAZ1B, LENG8, CTDSPL2, ZNF275, ZFAT, DESI2, MAP3K11, EPHA8, SORBS2, GNG2, L3MBTL3, CCDC85C, ANK1, CELF5, PARP8, EZH2, FAM169A, SOX4, KANK1, C6orf47, PPARGC1A, WWC1, DCUN1D4, KDM5A, PTP4A1, GATAD2A, MOCS1, EFNB3, HK1, USP47, PWWP2A, LMAN1, RAVER1, RELN, AFF3, VEZF1, VPS26A, RPS6KA1, PSMF1, NAPG, ATP11C, ROCK2, ZMYND11, ATCAY, MFAP3, MGAT5B, ZNF292, THRAP3, LHFPL3, PHOX2B, LSM14A, GPR158, PCGF3, SLC39A9, SEMA4C, KIF26A, SOBP, ASXL3, NEBL, JMJD1C, SLC22A23, CLMP, TMEM38B, SOGA1, JOSD1, UNC5D, ZNF607, CDR2L, TET2, ING1, PRPF40A, TMOD2, KIAA0930, ACVR2B, AHDC1, SZRD1, RHBDL3, KMT2C, DENND1A, GALNTL6, VPS37A, NOVA1, HIF1AN, TEAD1, FRMD4A, SLC20A1, XPR1, NBEA, FAM210B, IMPAD1, TULP4, APPBP2, PPM1L, SPEG, PAPPA, TRPS1, SCN8A, MTF2, ZNF148, ZEB2, SMG1, TSR1, ARFGEF2, AHCYL2, TAOK1, CNOT6L, PPARGC1B, RIMS3, ILDR2, KIAA1958, MLXIP, CHD6, GGCX, GAS7, ZBTB44, SCAI, PHF21A, LIMCH1
miR-1-3p	CORO1C, SMIM14, ARPC3, TAGLN2, GJA1, SERP1, TMSB4X, MMD2, BDNF, SLC10A7, G6PD, SRI, GLCCI1, CDK14, C5orf51, RIT2, ARCN1, KCNIP3, TMEM243, POGK, SLC44A1, EBPL, MMD, SRSF9, ANKRD29, UST, ANXA2, NDRG3, IGF1, MAB21L1, NXT2, GPD2, C9orf152, PAX3, RABGAP1L, TPM4, MAL2, MXD1, RAB5A, TMEM178A, TTR, GPR137C, E2F5, LASP1, C2orf69, CCSAP, HNRNPU, TWF1, SPRED1, GCH1, TSPAN4, EDN1, MSANTD2, TRIM2, VAMP4, PAX7, TNKS2, PPIB, GPR6, CAAP1, PIRT, PDCD10, ABHD2, BSCL2, CITED2, TDP1, NCL, SH3BGRL3, UNC119B, CHSY1, CCND2, FBXL14, TACR1, WDR48, CDC42SE1, AZIN1, CLTC, ZNF281, RAB43, HOXB4, MAN1C1, NANP, CPED1, SLC25A30, AP1G1, CNN3, SMARCB1, ADPGK, PGD, PDE7A, ZNF580, TIMP3, TPM3, RNF138, NFATC2, DDX5, ZFP36L1, RASA1, KIF2A, HMGN1, UBE2H, MON2, YWHAZ, TMCC1, FOSB, MPP5, RRBP1, LRCH1, CTTNBP2NL, HS3ST3B1, BAG4, SLC29A3, STC2, ZMAT3, RARB, YWHAQ, EIF4E, PTBP1, SNAP25, XPO6, OSBPL7, ABCB7, HELZ2, RNF141, HACE1, ISY1-RAB43, KTN1, PDGFA, MATR3, ARF3, PHAX, HSPD1, FBXO33, DHX15, RSPO3, CLCN3, THBS1, KMT2E, SLC25A22, MBLAC2,

	CPLX2, MEOX2, PTPRG, CPEB1, TPPP, KALRN, FAM155A, ANXA4, PFN2, BACH2, ETS1, SH3GL1, SEC63, KANK4, WDR1, MAP4K3, RSBN1L, DGKE, FUBP1, USP33, AXL, CLOCK, SRSF1, CD164, ZNF280C, PPP4R2, ARFIP1, HNRNPA3, MIER1, WBP1L, ANP32E, MECOM, NME1-NME2, FN1, CBL, OTX2, NINJ1, LRRC8A, COL25A1, PRKACB, PDE12, CREBL2, PDIK1L, SLC35B4, SLC39A9, SLC39A10, ATP6V1A, TGIF2, FAM83A, PICALM, C2CD5, SKIDA1, SNAI2, JARID2, STARD7, H3F3B, MET, SP2, FAM102A, CASK, LCOR, DGKH, HNRNPK, CXCR4, DCAF12L1, FBXW7, ATG13, FNBP1L, OSTF1, SOX9, ACAP2, COL4A3BP, ZNF800, PTMA, NFATC3, SYT1, SLC25A25, STX6, TRANK1, TGIF1, PIK3C2A, TBC1D15, NR4A2, RSBN1, RAB11FIP1, PDCD4, SEC62, TMEM135, TCF7L2, FAM91A1, KLF4, TNPO2, SLC2A13, MPZ, MYOCD, KCNJ2, ADAM12, ZFP36L2, AKAP11, PHF6, PLEKHO2, ELF1, ACTB, DLG4, RYBP, SRGAP2, PRKCE, ZNF24, UTRN, EAF1, RAP1B, CTBP2, ZBTB21, KRAS, RNF145, CAPRIN1, BTAF1, PCDH17, ARHGAP21, UBR5, SLC8A1, HAND2, NBEA, MTSS1, HDAC4, EYA4, EMX2, COL4A3, TAOK3, LEF1, SMARCC1, ATF2, SRXN1, TRPS1, ATXN1L, SLC30A9, KAT6A, PTPN1, PNP, SULF1, KAT6B, MBTD1, RNF38, GTF2B, GLIS2, GPR158, SEMA6D, COL19A1, GPR85, FAM168A, SPTLC3, GOLPH3, GIT1, MAP3K1, AP3D1, FRS2, VEGFA, FNDC3A, TTC7B, EML3, SLC15A2, TTC3, HIVEP3, PAX5, BCL7A, KNOP1, HIPK3, SLC8A2, WASL, TBX18, MNT, KLHL15, NOTCH3, PTAR1, FAM84A, FGD4, FNDC3B, SAMD8, UNC50, ANKRD23, FOXP1, JOSD1, AMOT, THRB, TSPYL4, ZMYND8, NFAT5, GNE, DLG2, STAG2, RALGAPA1, MPP7, GLCE, SEC22C, STK39, CHST11, ZBTB4, FRAS1, EFNB2, UBN1, IGFBP5, GMFB, WNK3, HNRNPA1, CBX6, RNF165, CDK6, ZNF827, RICTOR, PAK3, UHMK1, ASXL3, NAMPT, SLC25A36, KDM2B, MAPK1, SLC7A2, CD2AP, RNF213, HP1BP3, BSN, ZNF236, DCP2, NCOA1, RORA, TEX2, CAMK1D, HMGCR, UBN2, ANKIB1, MAP1A, SH3PXD2B, WASF2, TNPO1, WIPF2, ADAR, SMG7, PAX6, SETBP1, SFRP1, MAML2, C6orf120, AVL9, PLAG1, OLFML2A, FAM126A, PLXNA4, UBE4A, PEAK1, PPP2R5A, MR1, IPMK, SOX6, PPM1L, DAAM1, DDX3X, SLC44A2, NRP1, TECPR2, CREB5, SOX5, SLC7A11, ABI2, SPEG, BICD1, EPC1, DICER1, NEDD4L, ANKRD13C, OSBP, HIVEP2, TRA2B, KCTD10, ARF4, HIC2, SPATS2L

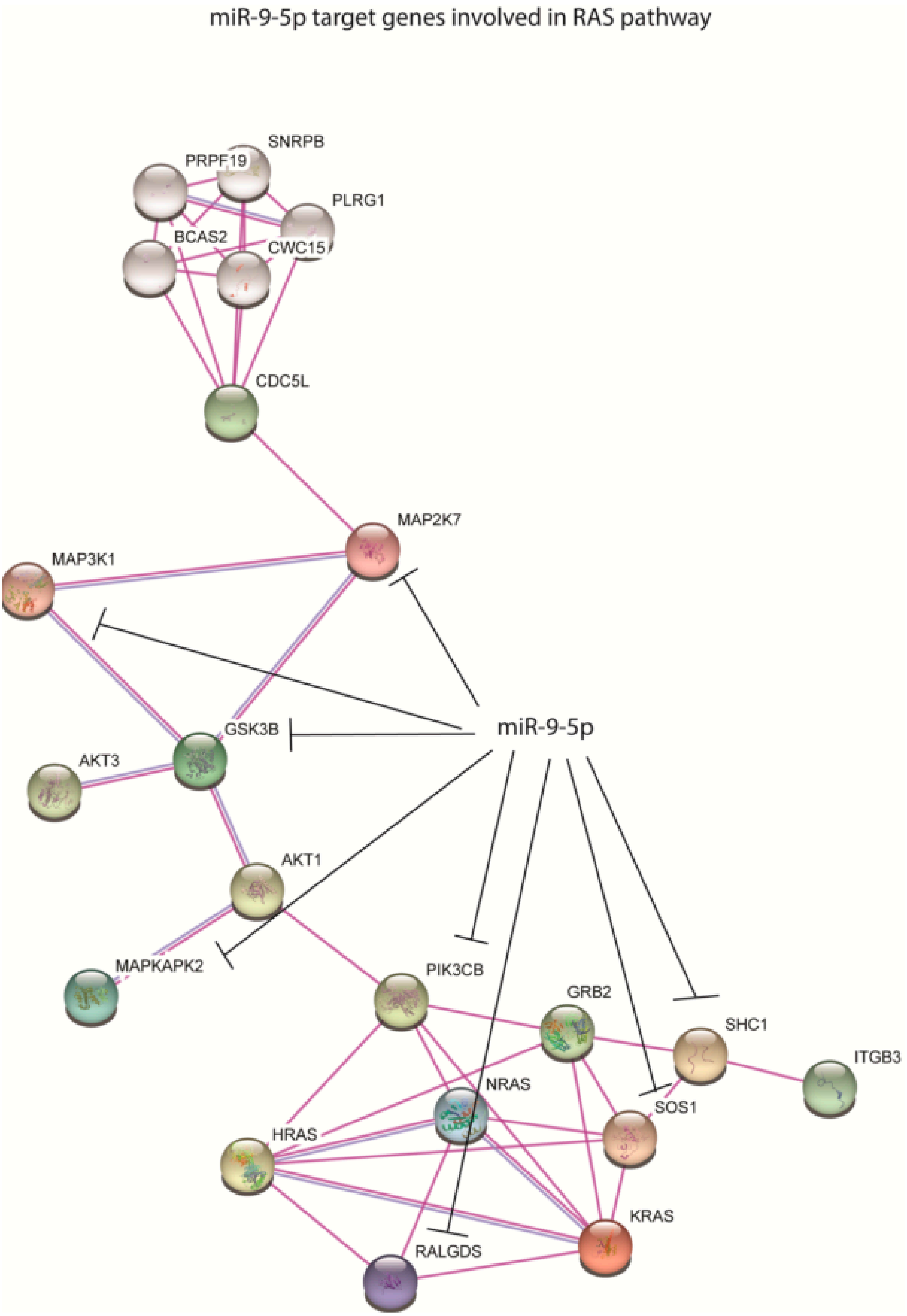

Supplementary Figure 1. Interconnections of proteins involved in the RAS pathway and regulated by miR-9-5p.

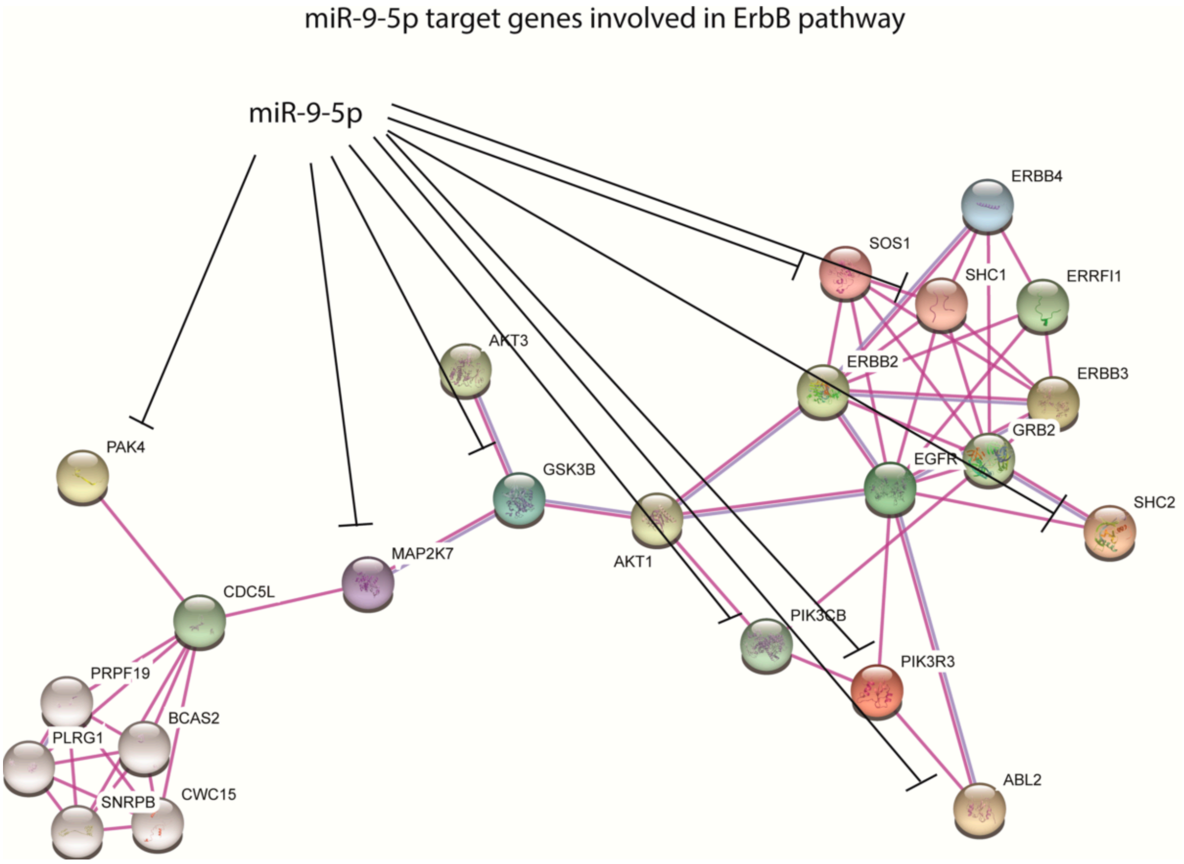

Supplementary Figure 2. Interconnections of proteins involved in the ErbB pathway and regulated by miR-9-5p.

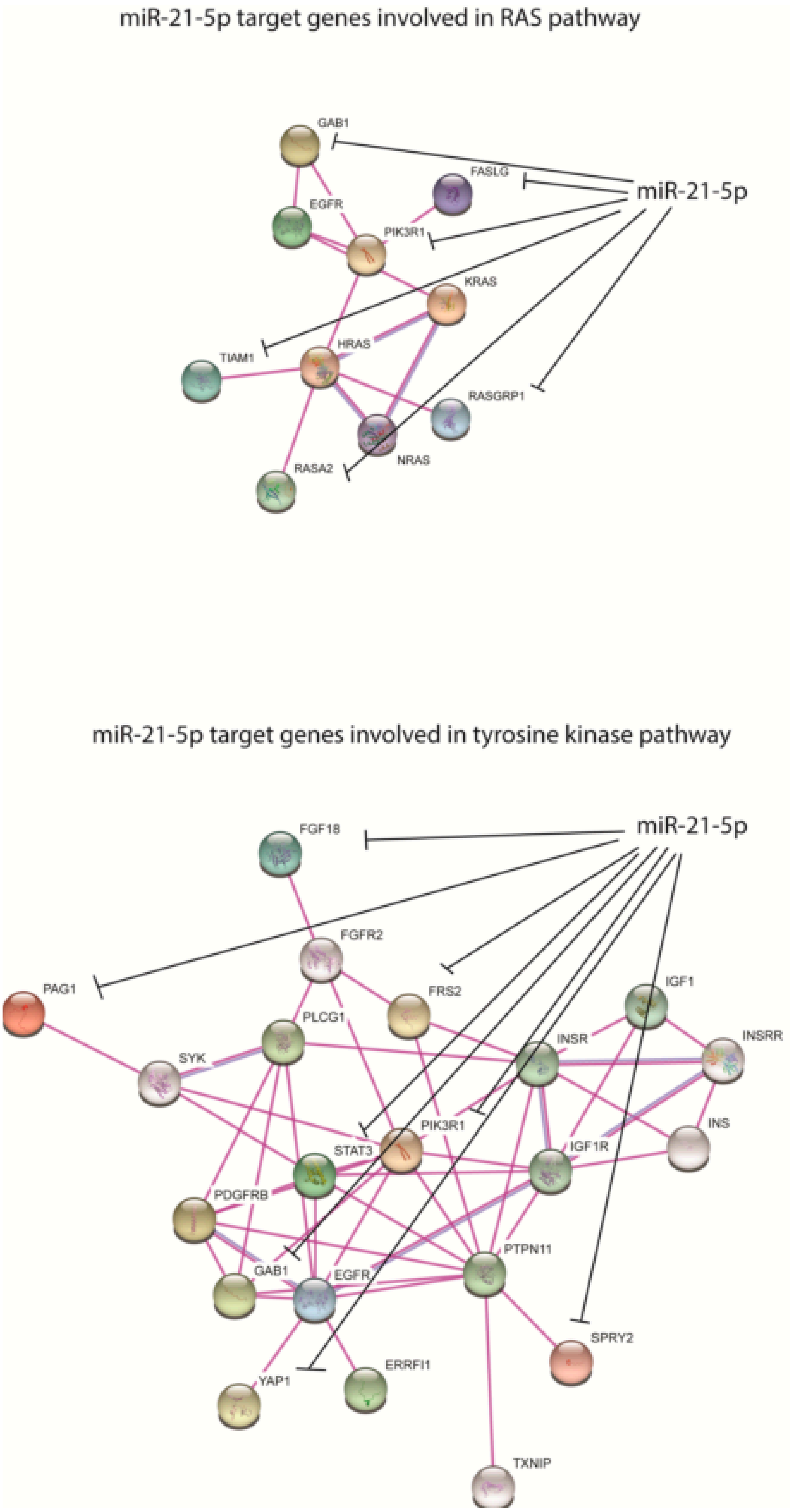

Supplementary Figure 3. The involvement of miR-21-5p in the RAS and tyrosine kinase pathway.

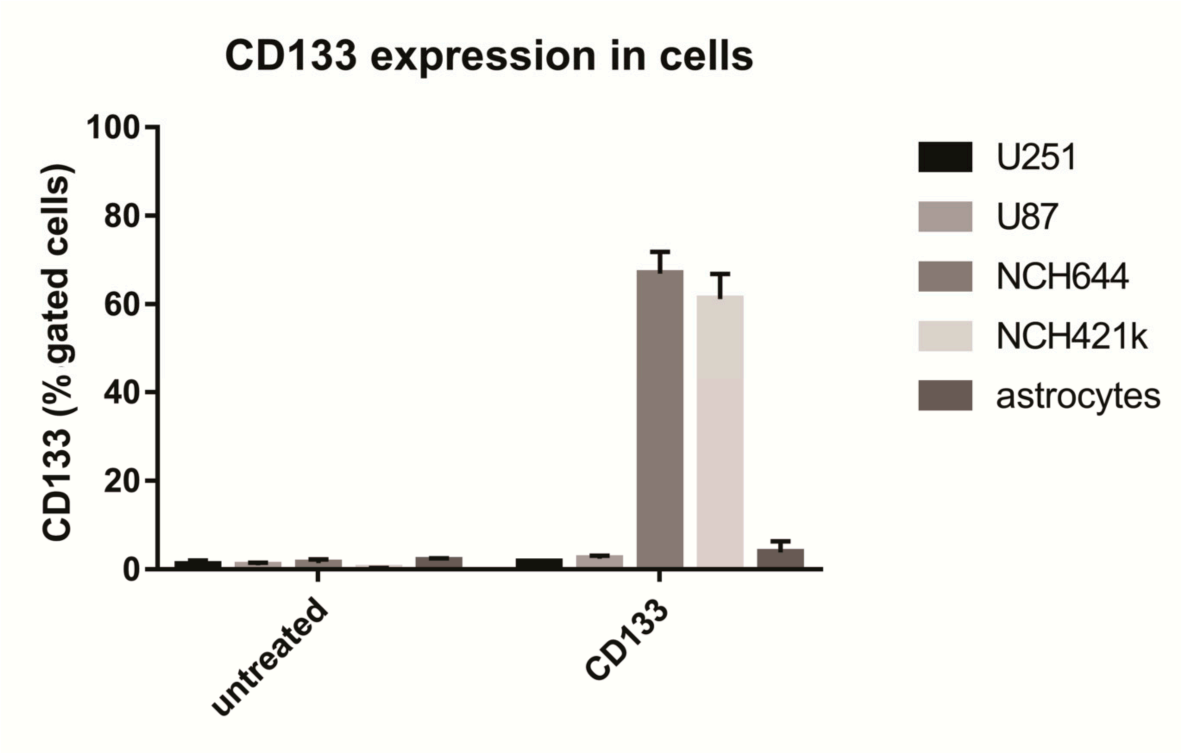

Supplementary Figure 4. Expression of CD133 on the surface of glioblastoma cells lines.
